# Stability of Selected Phenolic Acids Under Simulated and Real Extraction Conditions from Plants

**DOI:** 10.3390/molecules29245861

**Published:** 2024-12-12

**Authors:** Małgorzata Olszowy-Tomczyk, Łukasz Paprotny, Dorota Wianowska

**Affiliations:** 1 Department of Chromatography, Institute of Chemical Sciences, Faculty of Chemistry, Maria Curie-Skłodowska University in Lublin, Pl. Maria Curie-Skłodowska 3, 20-031 Lublin, Poland; 2 Research and Development Centre, ALAB Laboratories, ul. Ceramiczna 1, 20-150 Lublin, Poland

**Keywords:** chlorogenic acid, cynarin, plants extraction, MASE, UASE, PLE, SSDM

## Abstract

Currently, there is a significant demand for natural biologically active compounds. Emphasis is placed on improving the quality and safety of processed natural products, which is understandable in light of the frequently observed instability of natural compounds and their degradation, among others, to compounds of unknown biological activity. In this paper, the influence of typical conditions of currently used assisted extraction techniques on the stability of 5-O-caffeoylquinic acid and 1,3-di-O-caffeoylquinic acid during their simulated and real extraction from plants was investigated. In the experiments, extraction assisted by microwave radiation, ultrasound and pressure in procedures known as MASE, UASE and PLE techniques, respectively, was used. By comparing the amounts of native plant components, i.e., compounds present in the extract obtained, as shown, by the non-destructive SSDM technique with the amounts of these compounds estimated in extracts obtained by the above-mentioned techniques, it was proven that their content is variable. These differences are a consequence of two opposing processes, i.e., the success of the isolation process (its efficiency) and the degree of degradation/transformation of the main components. The results of the studies presented here can reduce the share of the second of the above, and consequently contribute to more effective obtaining of phenolic compounds from plants.

## 1. Introduction

Plants synthesize a huge number of organic compounds, among which the group of secondary metabolites (SMs) is worth noting. These compounds were long considered waste products that did not perform any important functions in the plant. However, not only have the enormous complexity of the SMs biosynthesis process and the participation of specific enzymes in each of the many pathways of their synthesis been demonstrated, but also their highly diversified biological activity [1]. It is because of a number of characteristic biological properties, both proven and suggested, that SMs are currently enjoying great interest in various industries and the scientific community.

An important component of vegetables, fruits, herbs or cereals are polyphenolic compounds that affect the functioning of the human body. Many of them stimulate the immune system, exhibit antioxidant activity and are characterized by anti-inflammatory, antibacterial, antifungal, antiviral, hepatoprotective and choleretic effects [2,3,4,5,6]. These also include compounds with anticancer activity and/or mitigating the side effects of chemotherapy [7]. However, currently not only is the pharmaceutical industry interested in the use and analysis of plant secondary metabolites, many of them are intentionally introduced into food and cosmetic products, enhancing their nutritional (nutraceuticals) and care (cosmeceuticals) effects. Others serve as markers in the quality control of various groups of natural products [5].

Due to the increased demand for SMs and interest in natural products with higher SMs content, more efficient methods of their isolation are being sought [8,9,10]. These activities are intensified by the fact that for many of them, chemical synthesis is an unprofitable undertaking, among others, due to the diversity and complexity of their structure. More accurate methods of their analysis are also being sought [11,12,13]. The essence of the issues raised is best explained by the chemical instability of many SMs. As a result, during isolation from plant material, they may be transformed/decomposed into other compounds, often with unknown biological effects on the human body, and the results of their analysis in plants may be erroneous. Therefore, it is not surprising that research on the stability of SMs is one of the important contemporary research trends.

**Figure 1:**
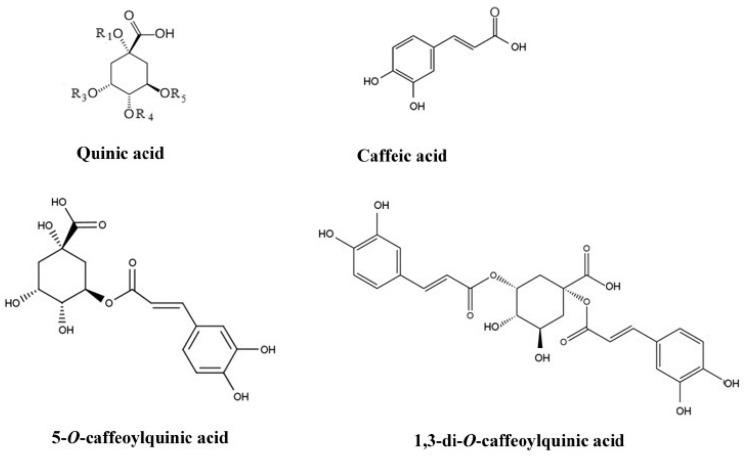
Exemplary structures of mono- and di-caffeoylquinic acids.

Chlorogenic acids (interchangeably called caffeoylquinic acids, CQAs) are ubiquitous and highly diverse polyphenolics with valuable health-promoting properties. From a chemical point of view, CQAs are a large family of esters formed between quinic acid and one or more *trans*-hydroxycinnamic acid derivatives. Figure 1 and Table 1 show exemplary structures of phenolic acids and their more complex depside forms, i.e., quinic and caffeic acids and caffeoylquinic and 1,3-di-O-caffeoylquinic acids, respectively. It should be added that, in addition to caffeic acid, depside forms with quinic acid can be formed by a number of other acids, such as p-coumaric and ferulic acids, yielding mono-, di- and tri-coumaroylquinic and feruloylquinic derivatives, respectively, and even more complex mixed structures.

There are many papers in the literature devoted to the isolation and analysis of CQAs in various plant matrices [13,14,15]. Similarly, much is known about their instability, especially thermal instability [16,17,18]. However, there is a lack of comparative studies of the stability of these compounds in various, current commonly used assisted extraction techniques such as microwave-assisted solvent extraction (MASE), ultrasound-assisted solvent extraction (UASE) and pressurized liquid extraction (PLE). With this in mind, in this study it was decided to check the influence of the so-called supporting parameters, i.e., those improving the isolation process and characteristics of the MASE, UASE and PLE techniques, on the stability of selected representatives of mono- and di-caffeoylquinic acids, using 5-O-caffeoylquinic acid (chlorogenic acid, 5-CQA) and 1,3-di-O-caffeoylquinic acid (cynarin, 1,3-diCQA), respectively. As a comparative method, in accordance with the results presented in [16,19], the SSDM technique was used. Stability was studied in model systems using standards of both compounds and under real conditions of their extraction from plants. In this part of the study, plant material was selected that varied in terms of plant organs (flowers, leaves, fruits or the whole plant as an herb), their hardness (soft flowers and hard unroasted coffee beans) and the qualitative and quantitative composition of compounds, including phenolic compounds, i.e., yarrow and coltsfoot herb, chamomile and tansy flower, leaves and the “heart” of artichoke inflorescence buds and green coffee beans. It is worth adding that some of these matrices are particularly related to chlorogenic acids. This applies to the artichoke, from which the Latin name (*cynara*) is derived the name of its characteristic component, cynarin, and to coffee beans, for which the content of chlorogenic acid is an indicator of quality.

## 2. Results and Discussion

### 2.1. Studies on the Stability of 5-CQA and 1,3-diCQA Acids in Simulated MASE Extraction Conditions—Identification of Derivatives

**Figure 2:**
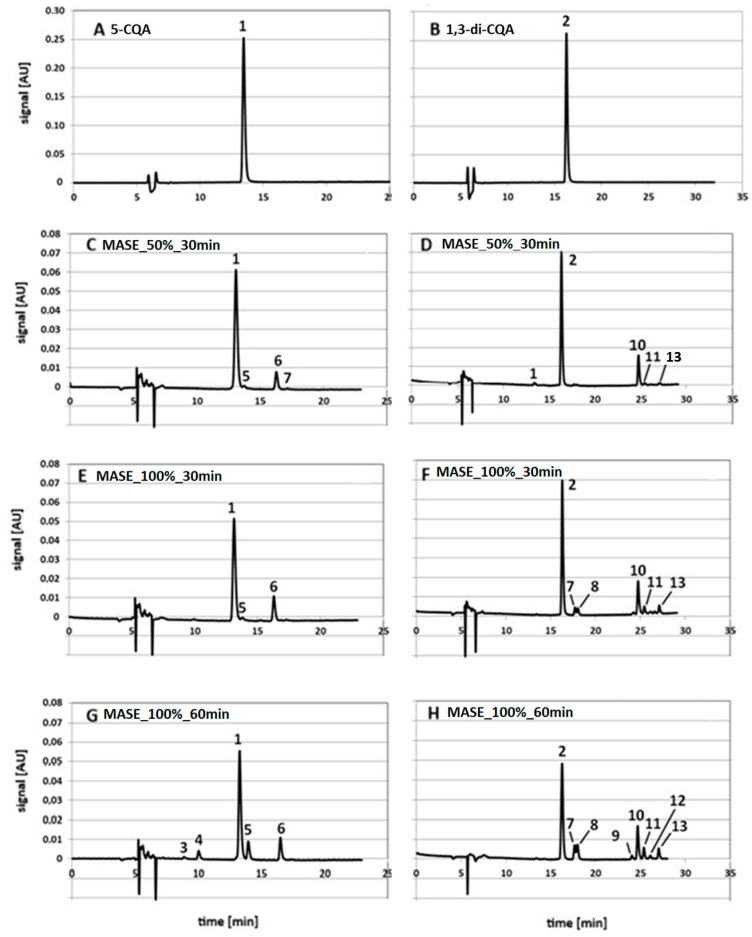
Representative chromatograms of 5-CQA (**A**) and 1,3-diCQA (**B**) standard solutions without and after simulated MASE performed for 30 min at 50% of the microwave generator power (**C**,**D**); 30 min at 100% microwave power (**E**,**F**) and 60 min at 100% microwave power (**G**,**H**) (detailed description in the text).

Exemplary chromatograms of 5-CQA and 1,3-diCQA standard solutions prepared in 60% MeOH in water, untreated and treated with microwave radiation, under model extraction conditions simulating the isolation of these compounds from plants using the MASE technique, are presented in Figure 2. Parts A and B of this figure show the chromatograms of the 5-CQA standard solution (peak no. 1, retention time 13.1 min) and 1,3-diCQA (peak no. 2, retention time 16.2 min), respectively, untreated with MASE. The following parts of the figure, show the chromatograms of 5-CQA and 1,3-diCQA standard solutions after the MASE process performed for 30 min at 50% of the microwave generator power (C and D); 30 min at 100% microwave power (E and F) and 60 min at 100% microwave power (G and H). The system chromatograms are presented in the Supplementary Materials.

As can be seen from the presented chromatograms, methanol–water solutions of 5-CQA and 1,3-diCQA standards after exposure to microwave radiation in the so-called simulated extraction conditions, in addition to the parent compound, contain a number of other substances. The presence of these compounds indicates that under the applied MASE conditions, both standards are decomposed/transformed into derivatives, and the number of derivatives formed and their content in individual extracts changes with the change in conditions of the simulated MASE process. In general, the chromatograms with fewer peaks are those obtained at 50% of the generator power after 30 min of exposure to radiation (see chromatograms C and D in the figure below). Extending the exposure time to microwaves and/or increasing the generator power leads to an increase in the number of derivatives formed under these conditions.

Among the degradation/transformation products of the 5-CQA and 1,3-diCQA standards visible in the chromatograms, the following substances were identified and ranked in the order of their elution in the RP-HPLC system:

- 1-O-caffeoylquinic acid (1-CQA, t_R_ = 8.5 min, peak no. 3);
- 3-O-caffeoylquinic acid (3-CQA, 9.9 min, peak no. 4);
- 5-CQA (peak no. 1, see Figure 2D);
- 4-O-caffeoylquinic acid (4-CQA, 13.7 min, peak no. 5);
- *cis* isomer of 5-O-caffeoylquinic acid (*cis*-5-CQA, 16.3 min, peak no. 6);
- caffeic acid (CA, 17.3 min, peak no. 7);
- caffeic acid methyl ester (CME, 17.6 min, peak no. 8);
- 3,4-O-dicaffeoylquinic acid (3,4-diCQA, 23.7 min, peak no. 9);
- 1,5-O-dicaffeoylquinic acid (1,5-diCQA, 24.7 min, peak no. 10);
- 3,5-O-dicaffeoylquinic acid (3,5-diCQA, 25.3 min, peak no. 11);
- *cis* isomer of 1,5-O-dicaffeoylquinic acid (*cis*-1,5-diCQA, 25.6 min, peak no. 12); and
- 4,5-O-dicaffeoylquinic acid (4,5-diCQA, 26.9 min, peak no. 13).

All these compounds are well described in the literature. In this study, the following observations were helpful in the identification:

- the *m*/*z* value for compounds 3, 4, 5 and 6 is the same as the *m*/*z* value of *trans*-5-*O*-CQA acid (compound no. 1), equal to 353, while for compounds 9, 10, 11, 12, 13 and 14 it is the same as the *m*/*z* value of *trans*-1,3-diCQA acid (compound no. 11) equal to 515 [20,21];
- in the RP-HPLC system, the order of elution of CQA and di-CQA is as follows: 1-CQA (peak no. 3) → 3-CQA (peak 4) → 5-CQA (peak 1) → 4-CQA (peak 5) and 1,3-diCQA (peak 2) → 1,4-diCQA (peak 14) → 3,4-diCQA (peak 9) → 1,5-diCQA (peak 10) → 3,5-diCQA (peak 11) → 4,5-diCQA (peak 13) [22,23];
- in the RP-HPLC system, cis-isomers have a longer retention than the corresponding trans forms [7,24];
- in the spectrum of cis-isomers, the absorption maximum is observed at shorter wavelengths of electromagnetic radiation compared to the spectrum of trans derivatives [24];
- the signal of cis-isomers in the chromatogram increases after the extension of the exposure of the 5-CQA and 1,5-diCQA standard solutions to UV–VIS [25].

The correctness of the identification was confirmed by the analysis of the caffeic acid and 1,5-diCQA standards. The data used to identify the degradation/transformation products of trans-5-CQA and trans-1,3-diCQA are summarized in Table 2. In order to gather all derivatives in one place, 1,4-O-dicaffeoylquinic acid (1,4-diCQA) is additionally included. This compound was present in the cynarin solution after the simulated UASE process which will be presented in the discussion below.

### 2.2. Assessment of the Amount of 5-CQA and 1,3-diCQA and Their Derivatives Under Simulated MASE Conditions

**Figure 3:**
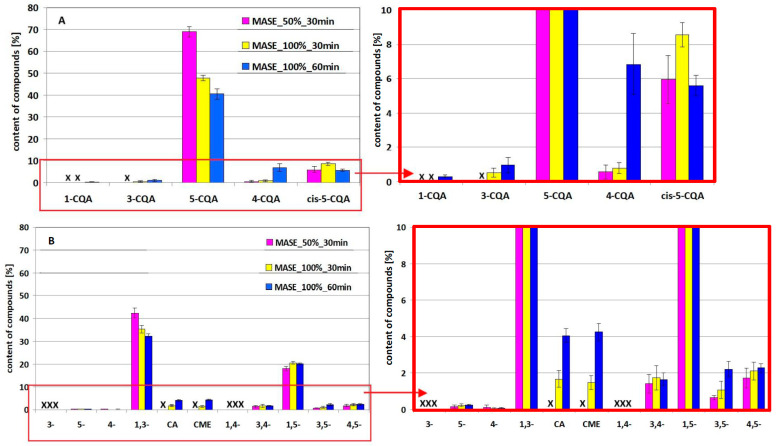
Effect of the microwave radiation generator power (50% or 100%) and exposure time (30 or 60 min) on the amounts of compounds revealed in the methanol–water solution of the 5-CQA standard ((**A**) and its enlarged fragment next to it) and the 1,3-diCQA standard ((**B**) and its enlarged fragment next to it) after simulated MASE extraction (data presented according to the elution order).

The results of the evaluation of the effect of exposure time and microwave radiation generator power on the amounts of 5-CQA and 1,3-diCQA derivatives revealed by the LC-PDA technique in methanol–water solutions of standards after the simulated extraction process using the MASE technique are presented in Figure 2 and Figure 3 for 5-CQA and 1,3-diCQA, respectively. The tests were conducted at 50% and 100% of generator power during a 30 min exposure to radiation and during a 60 min exposure at 100% microwave generator power. The obtained results are presented in Part A of each figure. In Part B, for the clarity of the results presentation, an enlarged fragment marked with a frame is presented. The “x” in each figure indicates the absence of a given derivative. The results of one-way analysis of variance are presented in Tables S1 and S2 for 5-CQA and 1,3-diCQA, respectively (see Supplementary Materials).

The data presented in the figures above, supported by statistical analysis (Tables S1 and S2), can be considered in two ways, i.e., in the context of the loss of the tested substances (5-CQA and 1,3-diCQA) and in the context of the types and amounts of derivative compounds formed. In the first case, a comparison of the amount of 5-CQA and 1,3-diCQA remaining in solution after simulated extraction, approximately 70% and 40%, respectively (see Figure 3A), shows the following:

- 5-CQA is more stable than 1,3-diCQA under the same process conditions;
- changing the MASE conditions differentiates the loss of both compounds;
- increasing the power of the microwave generator, with the same exposure time of the standards, causes their greater transformation/degradation;
- extending the exposure time from 30 to 60 min at a given generator power (in this case 100% power) also causes greater losses.

In both cases, i.e., after changing the generator power and extending the exposure time, a greater loss of 5-CQA is observed than that of 1,3-diCQA (compare the differences in the height of the 5-CQA and 1,3-diCQA bars in Figure 3A). This conclusion is confirmed by statistical analysis, showing statistical significance of the effect of power and time for 5-CQA (high values of the Fisher coefficient (*F*)), and statistical significance only of the generator power effect for 1,3-diCQA (22.2 < *F*_5-CQA_ < 196.1, 6.8 < *F*_1,3-diCQA_ < 19.4, *Fcrit* = 7.7).

As for the type and amount of individual derivatives formed from both parent compounds, changing conditions leads to an increase in the number/amount of derivatives. In the case of 5-CQA, after 30 min of exposure of the standard to 50% of the generator power, 4-CQA and *cis*-5-CQA appear, with the amount of the second derivative being greater (see Figure 3B). It may be surprising that the dominant derivative under MASE conditions is the *cis*-isomer. Nevertheless, microwave photochemistry is a new, rapidly developing scientific discipline combined with photocatalysis, among others, in synthetic chemistry [26]. This discipline is often, but not necessarily, based on the use of a special electrodeless discharge lamp that emits UV–VIS radiation under the influence of microwave radiation. Chlorogenic acids are known for their photochemical instability. Therefore, most likely in the case of 5-CQA, a 30 min exposure to the light of a bulb illuminating the interior of a switched-on microwave oven is already sufficient to initiate photoisomerization at 50% of the generator power. Increasing the power and extending the time lead to a marked increase and then decrease in the amount of *cis*-5CQA. In the case of 4-CQA, increasing the power and, in particular, extending the exposure time leads to a significant increase in the amount of this derivative. At higher power, the 3-CQA isomer appears successively (its amount increases with exposure time) in addition to the 1-CQA isomer.

In the case of 1,3-diCQA derivatives (see Figure 3B), after 30 min of exposure to 50% generator power, 3,4-, 3,5-, 4,5- and 1,5-diCQA appear in the extract, with the amounts of 3,4- and 4,5-diCQA being at a comparable level of about 2% of the original amount of 1,3-diCQA, while the amount of 1,5-diCQA is close to 20% of the original amount of the 1,3-diCQA standard. In addition to the above transformation products, degradation products appear in the extract, i.e., mono-caffeoylquinic acids (5-CQA and 4-CQA) and caffeic acid and its transformation product—caffeic acid methyl ester (CME). Increasing the exposure time at 100% generator power leads to a statistically insignificant increase in the amounts of 3,4-diCQA, 1,5-diCQA and 4,5-diCQA (*F* < *Fcrit*) and a statistically significant increase in 3,5-diCQA (*F* > *Fcrit*). The presence of 3-CQA was not confirmed in the extracts. Nevertheless, it is possible that this derivative is present but below the detection limit of the method. Similarly, the presence of 1,4-diCQA and the 1-CQA isomer, not included in Figure 3B, was not confirmed.

### 2.3. Stability/Quantity Studies of 5-CQA and 1,3-diCQA and Their Derivatives Under Simulated UASE Extraction Conditions

**Figure 4:**
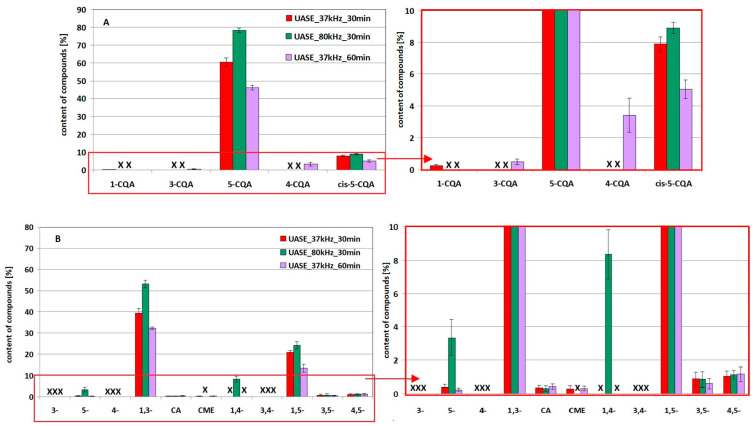
Effect of ultrasound frequency (37 kHz or 80 kHz) and exposure time (30 min and/or 60 min) on the amounts of compounds revealed in the methanol–water solution the 5-CQA standard ((**A**) and its enlarged fragment next to it) and the 1,3-diCQA standard ((**B**) and its enlarged fragment next to it) after simulated UASE extraction (data presented according to the elution order).

The quantitative evaluation of 5-CQA and 1,3-diCQA residues after the simulated UASE extraction process along with the amounts of derivatives formed under these conditions are shown in Figure 4A,B, for 5-CQA and 1,3-diCQA, respectively. In the figure, as before, to facilitate the interpretation of the data, the fragment marked in parts A and B of the figure is enlarged next to it. The results of one-way analysis of variance are presented in Tables S3 and S4. Exemplary chromatograms of 5-CQA and 1,3-diCQA standards solutions exposed to ultrasound at 37 kHz and 80 kHz for 30 min and/or 60 min in the simulated UASE process are shown in Figure S1 (see Supplementary Materials).

Analysis of the obtained results allows us to conclude that exposure of chlorogenic acids to ultrasound leads to their transformation and/or degradation. The effectiveness of these processes depends on the frequency and time of exposure to ultrasound. As a result, changing the UASE conditions varies not only the amounts of 5-CQA and 1,3-diCQA remaining in solution after the process, but also the number and content of the resulting derivatives.

Comparing the amounts of 5-CQA and 1,3-diCQA estimated in solutions after the simulated UASE process performed for both compounds under the same conditions (see Figure 4, in the figure the missing compound is marked with “x”), it can be stated that overall a lower loss of 5-CQA was observed. Thus, under the UASE conditions (similar to MASE), 5-CQA is more stable compared to 1,3-diCQA. Reducing the frequency and/or prolonging the exposure generate a greater loss of both compounds, which is accompanied by the appearance of a variable number/amount of transformation/degradation products. The dominant 5-CQA derivative, regardless of the conditions used, is the *cis*-5-CQA isomer (see Figure 4A, peak 6). The highest amount of this compound was found in solutions exposed for 30 min at 80 kHz (see chromatogram C in Figure S1), the lowest after 60 min of exposure to 37 kHz (see chromatogram E in Figure S1). In addition to the above-mentioned derivative, 1-CQA was identified in solution after a shorter exposure at 37 kHz (peak 3 in Figure S1). Longer exposure at 37 kHz resulted in the appearance of 3-CQA and 4-CQA, peaks 4 and 5 in Figure S1, respectively. The absence of 1-CQA in the extract after 60 min of exposure, in the face of a relatively large amount of 4-CQA, confirms the more destructive nature of the lower ultrasound frequency. High values of the Fisher coefficient obtained in the statistical analysis of the effect of frequency and time on the amounts of 5-CQA and *cis*-5-CQA, collected in Table S3, confirm the validity of the above conclusion.

Considering the content of individual 1,3-diCQA (cynarin) derivatives (see Figure 4B and Table S4), reducing the frequency and increasing the exposure time of the standard generally leads to a decrease in the amount of derivatives. Thus, during the process, not only are new compounds formed as a result of the transformation of the parent molecule, but the derivatives are also degraded. Statistically significant changes in the quantities were observed for 5-CQA and 1,5-diCQA derivatives (*F* > *Fcrit*, see Table S4). The noticeable increase in the amount of 4,5-diCQA with increasing time may suggest greater stability of this cynarin derivative. However, considering that derivatives are formed and transformed/degraded into other compounds at the same time, the validity of this conclusion is questionable, which is confirmed by statistical analysis (*F* < *Fcrit*). In the 1,3-diCQA solution after the simulated UASE process performed at a higher frequency for 30 min, the 1,4-diCQA content was estimated at 8%. The presence of this compound, as well as the higher 5-CQA content found under these UASE conditions, may further indicate the less destructive nature of the higher ultrasound frequency.

### 2.4. Stability/Quantity Studies of 5-CQA and 1,3-diCQA and Their Derivatives Under Simulated PLE Extraction Conditions

**Figure 5:**
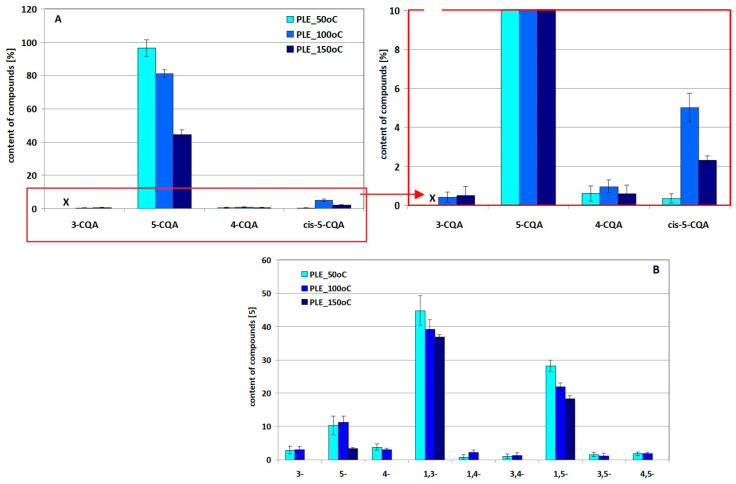
Effect of 50 °C, 100 °C and 150 °C temperatures under PLE conditions on the amounts of compounds revealed in the methanol–water solution of the 5-CQA standard ((**A**) and its enlarged fragment next to it) and the 1,3-diCQA standard (**B**) standards (data presented according to the elution order).

Figure 5 summarizes the quantities of 5-CQA and 1,3-diCQA remaining after a simulated 10 min PLE extraction process at 50 °C, 100 °C and 150 °C, along with an assessment of the quantities of derivatives obtained under these conditions from both standards. The results of the one-way analysis of variance are gathered in Tables S5 and S6, separately for 5-CQA and 1,3-diCQA, respectively. Exemplary chromatograms of methanol–water solutions of 5-CQA and 1,3-diCQA standards after the simulated PLE process are presented in Figure S2.

The presented research results prove the instability of 5-CQA and 1,3-diCQA also under simulated PLE process conditions. However, while for the 5-CQA standard no qualitative differences in derivatives are observed compared to those identified in the solution after the MASE and UASE processes (compare the chromatograms in Figure 2, Figures S1 and S2), for 1,3-diCQA a larger number of derivatives is typically visible, eluting after 21 min of analysis. This fact is not surprising, since the evaluation of the effect of the two previously discussed assisted extraction techniques on the stability of both standards showed a greater instability of the dicaffeoylquinic acid representative. The identification of these new derivatives is not yet complete. However, in order to demonstrate that the larger number of 1,3-diCQA derivatives is not a matter of chance and results, among others, from insufficiently cleaned sand used to fill the extraction vessel or insufficient washing of residues of previous extracts from the system, but a consequence of the PLE process conditions, simulated 10- and 20-min extractions of cynarin were performed using water as the extractant. The results of this experiment are presented in Figure S3 in the Supplementary Materials.

Analysis of the data in Figure S3, in addition to the similarities to the elution pattern seen in chromatograms B and D in Figure S2, allows us to state the following:

- there is an increase in the 5-CQA derivative signal (compare peak height 1 in Figures S2D and S3A);
- there are decreases in the 5-CQA, 1,3-diCQA and 1,5-diCQA signals with increasing extraction time;
- there are increases in the 3,4-diCQA and 4,5-diCQA signals with increased extraction time; and
- there appear 3-CQA and 4-CQA derivatives.

All these observations indicate a different degree of cynarin transformation under PLE conditions. The validity of this conclusion is confirmed by the estimated content analysis of individual 1,3-diCQA derivatives presented in Figure 5B and supported by statistical analysis (Table S6). In light of the presented research results, cynarin under PLE conditions, unlike the previously presented transformation/degradation products detected in solutions after simulated MASE and UASE extraction, reveals the full spectrum of transformation products into positional isomers of diCQA and mono-CQA not only at 50 °C but also at 100 °C. This fact, however, may indicate a less destructive nature of the PLE process, since in the case of the other techniques not all of these derivatives were found in one extract. This is also evidenced by the generally larger amounts of 5-CQA and 1,3-diCQA and lower contents of derivatives estimated in solutions after the PLE process.

### 2.5. Studies on the Stability/Quantity of 5-CQA and 1,3-diCQA and Their Derivatives Under Simulated SSDM Extraction Conditions—Comparison of the Destructive Nature of Assisted Extraction Techniques

In light of the results presented in [16,17], the number of derivatives generated in the SSDM process for the *trans*-5-CQA standard is very limited. However, there are no reports on the stability of cynarin under SSDM conditions. To fill this gap, the 1,3-diCQA standard was subjected to 10-min grinding with sand without the addition of dispersing liquid, implementing the so-called dry variant of the SSDM procedure and eluted using a 60% methanol in water solution [19]. To enable comparison, the stability of 5-CQA was tested under the same conditions of the SSDM procedure. Example chromatograms obtained in this series of studies are shown in Figure S4.

Analysis of the data presented in Figure S4 (see Supplementary Materials) allows us to conclude that while in the chromatogram of the cynarin solution after the simulated SSDM process (chromatogram A) there are no peaks of other substances visible apart from the peak of the parent compound (which confirms the stability of 1,3-diCQA), in chromatogram B, in addition to the 5-CQA peak, a *cis*-5-CQA peak is visible. The presence of this derivative, in view of the previously demonstrated tendency of 5-CQA to photoisomerize, is not surprising. The estimated amount of *cis*-5-CQA does not exceed 3%, which, compared to previously presented results (up to 10%), yields a good result, generally confirming the stability of chlorogenic acids under SSDM conditions.

Considering the demonstrated stability of the parent compounds under SSDM conditions, it was decided to use the content of compounds estimated after the SSDM process to compare their amounts in solutions after simulated MASE, UASE and PLE extraction. These data are summarized in Table 3. The results of statistical analysis showing the influence of the conditions of individual techniques on the losses of both compounds are compared in Table 4.

The analysis of the presented data, revealing lower contents of 5-CQA and 1,3-diCQA in solutions subjected to simulated MASE, UASE and PLE extraction compared to the amounts estimated after the SSDM process, confirms the destructive nature of the former. Comparison of F values from Table 4, although revealing the statistical significance of the differences, does not allow for an unambiguous ranking of techniques in terms of their destructive impact, i.e., the higher the Fisher coefficient value, the more destructive the nature of a given technique. Based on the changes in this value within a given technique, one can only conclude about the magnitude of the destructive impact of conditions/parameters, i.e., decomposition deepens with a decrease in the ultrasound frequency or an increase in the microwave generator power, process temperature and its duration. However, based on the number of derivatives formed, it is not possible to conclude about their stability because two opposing processes occur simultaneously—the formation of derivatives and their decomposition.

In summary, caffeoylquinic acids in model systems simulating their extraction by MASE, UASE and PLE techniques undergo degradation and transformation. Their stability depends, among others, on the number of substituted hydroxycinnamic acid derivatives in the quinic acid molecule, the process conditions specific to each technique and the temperature and time of exposure to the extraction aid. Regioisomerization is the dominant transformation pathway for both mono- and di-caffeoylquinic acids. The change in the quality and quantity of the formed isomers is consistent with the migration pathway of the cinnamoyl group known from the literature [22,23,24]. In the case of di-caffeoylquinic acids, in addition to the above-mentioned regioisomerization, hydrolysis to mono-caffeoylquinic acids and caffeic acid (quinic acid) and its transesterification take place.

### 2.6. Studies on the Stability/Quantity of Chlorogenic Acids and Their Derivatives in Real Extraction Conditions from Diverse Plant Matrices—Comparison of Acid Content in Extracts Obtained by MASE, UASE, PLE and SSDM Techniques

To compare the effect of assisted extraction techniques on the amounts of derivatives estimated for 5-CQA and 1,3-diCQA standards in model systems with the amounts of these compounds determined in real extraction conditions using MASE, UASE and PLE methods and to answer the question whether all identified compounds are native components of the plant matrix, another series of experiments was conducted. The research was carried out on various matrices, using representatives of plants belonging to the *Asteraceae* family, i.e., yarrow and coltsfoot herb, tansy and chamomile flowers, leaves and the “heart” of artichoke inflorescence buds, and green coffee beans. In these experiments, the SSDM technique was used as a comparison method.

**Figure 6:**
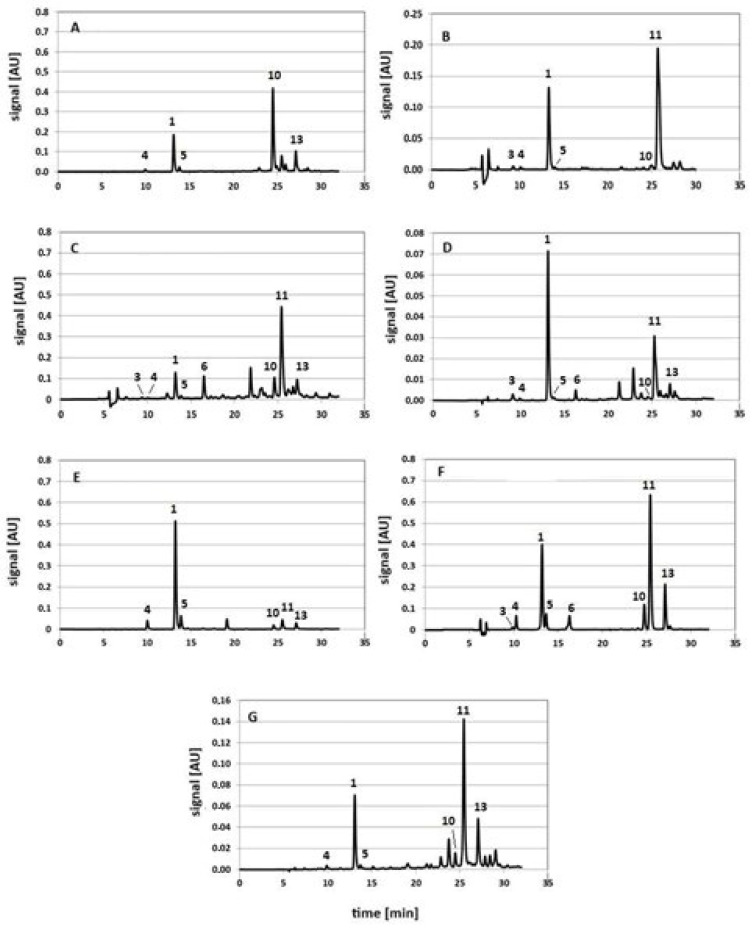
Representative chromatograms of extracts of coltsfoot (**A**), yarrow (**B**), chamomile (**C**), artichoke leaves (**D**), green coffee beans (**E**), artichoke bud “heart” (**F**) and tansy herb (**G**) obtained via UASE at 37 kHz.

Example chromatograms of extracts of the above-mentioned raw material, obtained using the UASE technique, are shown in Figure 6. The system chromatograms are presented in the Supplementary Materials.

The amounts of compounds estimated in individual extracts obtained under conditions analogous to those used in the model systems and expressed in µg/g ± SD are collected in Tables S7, S9, S11, S13, S15, S17 and S19 for coffee beans, artichoke leaves, artichoke hearts, coltsfoot, chamomile, tansy and yarrow, respectively. The last row of each of these tables shows the total amounts of compounds in the extracts. The ANOVA results of the effect of conditions/techniques on the amounts of compounds estimated using extraction techniques or the amounts revealed using the SSDM technique are presented in the corresponding tables, separately for each plant raw material (see Tables S8, S10, S12, S14, S16, S18 and S20). To facilitate analysis, statistically significant differences are marked in bold in each table. The analysis of the presented research results leads to the following conclusions:

- Compounds identified in model systems as degradation and/or transformation products of 5-CQA and 1,3-diCQA occur in real systems. Their number and content, however, varies and depends on the type of plant and its matrix, the extraction technique and the process conditions applied. An exception is CME identified in chamomile flower extracts under MASE and UASE conditions (see Table S16). Considering that this compound was not detected in the extract obtained using the SSDM technique, it should be considered an artifact.
- In general, the highest number of tested compounds was estimated for coffee (see Table S8), and the lowest for chamomile (Table S16). Artichoke (leaves) is characterized by the highest numbers of identified 5-CQA and 1,3-diCQA transformation/degradation products (Table S10). In the hearts of artichoke inflorescence buds, i.e., in the youngest elements of the plant, in contrast to artichoke leaves, the presence of CA and 1,4-diCQA was not confirmed (see Table S12). This fact can be related to the age of individual morphological elements of the plant (“aging” of the plant) and/or the storage time of the collected raw material.
- Among the mono-caffeoylquinic acids, the dominant compound in all plants is the 5-CQA isomer. The contents of 4-CQA and 3-CQA are lower, with the amount of 4-CQA exceeding the amount of 3-CQA. 1-CQA and cis-5-CQA are relatively rare compounds. Their presence was confirmed for artichoke, yarrow and tansy and coffee and artichoke, respectively.
- Among the di-caffeoylquinic acids, the most popular isomer is 1,5-diCQA and the rarest is 1,3-diCQA (this compound was identified only in artichoke extracts, it should be added that it is not a dominant compound although it is considered characteristic of this plant). Generally, 1,5-diCQA is the dominant representative of di-CQA acids. Only in the case of coltsfoot the dominant representative of this group of compounds is the 3,4-diCQA isomer.
- By comparing the amounts of native plant components, i.e., compounds present in the SSDM extract with the amounts of these compounds estimated in extracts obtained using assisted extraction techniques, it can be concluded that their content is a consequence of the success of the isolation process (its efficiency) and the degree of degradation/transformation of the main components during assisted extraction techniques. The greatest differences in the total amount of analyzed compounds, estimated in relation to the amounts revealed for each plant in the SSDM process, are observed for the MASE technique, UASE at an ultrasound frequency of 37 kHz and PLE conducted at 150 °C.
- When analyzing the contents of model compounds (mainly 5-CQA due to their common occurrence) with the amounts of their identified derivatives in extracts of individual plants, it is difficult to find unambiguous correlations. This may indicate differences in their metabolic pathways leading to the synthesis of other acids/compounds.

## 3. Materials and Methods

### 3.1. Materials

Acetonitrile (HPLC and MS grade), methanol and acetic acid were purchased from the Polish Chemical Plant POCh S.A. (Gliwice, Poland). Formic, caffeic acid, along with 1,3- and 1,5-*O*-dicaffeoylquinic acids, were purchased from Sigma Aldrich (Seelze, Germany). The *trans*-5-*O*-caffeoylquinic acid standard was supplied by Loba-Chemie Austranal Praparate (Austria). The sand used as abrasive material in SSDM was a donation from a local glassworks company. It was fractionated, leached with 1M HCl, washed out with distilled water to neutrality and dried. A 0.2–0.4 mm fraction was applied in the experiments. Water was purified on the Milli-Q system from Millipore (Millipore, Bedford, MA, USA).

The following plants were used in the study: flowers of tansy (*Tanacetum vulgare* L.) and chamomile (*Matricaria chamomilla* L.), herb of coltsfoot (*Tussilago farfara* L.) and common yarrow (*Achillea millefolium* L.), leaves and stems of artichoke (*Cynara scolymus* L.); fresh artichoke heart (*Cynara scolymus* L.) and green coffee beans. All were purchased from a local herbalist (Lublin, Poland). Most of the material was in a dried form. Only in the case of fresh artichoke heart was the material pre-dried in air at room temperature. Immediately prior to sample preparation, each plant material was ground with a Braun cutting mill to obtain the particle size of 0.2–0.4 mm and its precisely weighed portions extracted.

### 3.2. Extraction Methods Used During the Studies

Studies on the stability/quantity of selected representatives of secondary plant metabolites were carried out in simulated and real conditions of their isolation from plant material, using assisted extraction techniques MASE, UASE and PLE as well as the SSDM technique. A detailed description of the experiments is presented below separately for each of the extraction techniques used.

#### 3.2.1. MASE

Microwave-assisted solvent extraction was performed in a Plazmatronika UniClever BMZ I microwave oven (Wrocław, Poland) allowing for the variation of the radiation cycle time and generator power. Extractions were carried out on 0.2 g of plant samples or 10 mg of standards. The samples were quantitatively transferred to 60 mL (10 mL for standards) screw-on glass vials and poured with 45 mL of 60% methanol and extracted under the following conditions:

- microwave generator power: 50 and/or 100% of maximum power (800 W);
- extraction time: 30 and/or 60 min in a single extraction cycle regardless of the plant matrix and analyte.

To separate the extract from the plant material, centrifugation was used (10 min, 2700 rpm). After centrifugation, the extract was transferred to 50 mL volumetric flasks, which were filled to the mark with extractant. For statistical purposes, three independent extractions were performed for each type of sample (plant, standard) and process conditions.

#### 3.2.2. UASE

Ultrasound-assisted solvent extraction was carried out in an Elmasonic P ultrasonic bath. A weighed portion of the previously prepared plant material (0.2 g of plant portion of) or an appropriate standard (10 mg) was placed in a tightly screwed vial with a capacity of 60 mL (10 mL in the case of standards), and 45 mL of 60% methanol–water solution was added. The extraction process was carried out under controlled conditions as follows:

- frequency: 37 kHz and/or 80 kHz;
- ultrasonic generator power: 50% and/or 100% of the maximum power (720 W);
- extraction time: 30 or 60 min;
- temperature: 25 °C.

In order to separate the extract from the plant matrix, the whole sample was centrifuged (10 min, 2700 rpm), and the supernatant portions were transferred quantitatively to 50 mL volumetric flasks (25 mL in the case of simulated extraction of standards), the content of which was supplemented to the mark with the extractant and subjected to chromatographic analysis. For statistical purposes, three independent extractions were performed for each type of sample (plant, standard).

#### 3.2.3. PLE

The pressurized liquid extraction was performed on a Dionex ASE200 apparatus (Dionex Corp., Sunnyvale, California, USA). Portions of pretreated plant material (0.2 g) or standard (under simulated extraction conditions, 10 mg) were mixed with an inert material (sand) and placed in 22 mL stainless steel extraction vessels. The content of each vessel was extracted with 60% methanol–water solution under the following conditions:

- extraction time: 10 and/or 20 min;
- temperature: 50, 100 and 150 °C;
- pressure: 40 bar.

At the end of the process, the extraction vessel was rinsed with the extractant in a volume equal to 60% of the capacity of the empty vessel, and then purged with nitrogen at 150 psi for 60 s. The extracts were collected in 60 mL glass screw-cap vials with Teflon inserts, quantitatively transferred to 50 mL volumetric flasks, which were filled to the mark with the extractant. Between runs, the system was rinsed with extraction solvent. For statistical purposes, three independent extractions were performed for each sample type.

#### 3.2.4. SSDM

Portions of pre-ground plant material or standard (0.1 g of plant and 10 mg of standard, respectively) were placed in a glass mortar and mixed with sand using 0.8 g of dispersing material. The mortar content was ground with a glass pestle for 10 min until a homogeneous mixture was obtained. After homogenization, the mixture was transferred quantitatively to a 3 mL syringe barrel (SPE column) with a fitted filter paper disc placed at the bottom. After pressing the transferred material with the plunger of a syringe, the elution process was started into a 25 mL volumetric flask. For statistical purposes, three independent extractions were performed for each type of sample.

### 3.3. HPLC

Analyses were performed on a Dionex DX600 liquid chromatograph (Dionex Corp., Sunnyvaley, CA, USA) equipped with a Rheodyne automatic injection valve with a 25 µL loop, a GP50 gradient pump, a PDA100 detector and a Prodigy ODS-2 column (5 µm, 250 × 4.6 mm, Phenomenex, Torrance, CA, USA) with a pre-column from the same company. Component A of the mobile phase was 5% aqueous acetic acid solution. Component B was acetonitrile. Changes in the amount of B in the mobile phase were as follows: from 10 to 35% in the time interval 0–30 min, decreasing to the initial value (10%) in 2 min. Before the next analysis, the column was rinsed with the initial composition of the mobile phase for 10 min. The flow rate of the mobile phase was 0.5 mL/min. Separations were monitored at a wavelength of 330 nm. Spectra were collected in the range from 200 to 700 nm. Qualitative analysis was performed by comparing the retention times and UV–VIS spectra of the peaks in the chromatograms of the extracts with those obtained for the standards of the corresponding compounds. Quantitative analysis was performed by external calibration, based on the calibration equations obtained for five concentration levels of the calibration solutions of the appropriate standards. The values of the slopes (a), free terms (b) and regression coefficients (R^2^) of the obtained equations are presented in Table 5. In this analysis, the 5-CQA, 1,3-diCQA, 1,5-diCQA and caffeic acid standards were used. Due to the lack of 1-, 3-, 4- and cis-5-CQA standards, their contents were determined relative to the calibration curve obtained for 5-CQA. In the quantitative analysis of diCQA acids, the 1,3-diCQA and 1,5-di CQA calibration curves were used.

### 3.4. LC–MS

LC/MS analyses were performed on a system consisting of an LC-20AD chromatograph and an LCMS-8030 triple quadrupole mass spectrometer (Shimadzu, Kyoto, Japan) equipped with an ESI source. Separations were carried out on a Prodigy ODS-2 column (5 µm, 250 × 4.6 mm) using a gradient elution profile. Mobile phase A was 25 mM formic acid in water. Mobile phase B was 25 mM formic acid in acetonitrile. The gradient program started with 5% B, increased to 95% in 60 min and ended with an isocratic elution at 95% B for 10 min. The total time was 70 min at a mobile phase flow rate of 0.4 mL/min. MS spectra, in the range of 100–2000 *m*/*z*, were collected continuously during each measurement. ESI operating in negative polarity mode was carried out under the following conditions: voltage—3.5 kV, shielding gas—40 units, auxiliary gas—10 units, eluent gas—10 units and capillary temperature—320 °C. Nitrogen (>99.98%) was used as shielding, auxiliary and eluent gas. Secondary ion fragmentation (MS^2^) was used to identify chlorogenic acid derivatives. Collision energies for each tested compound were selected individually.

### 3.5. Statistical Analysis

The results of the quantitative analysis are presented as the mean value ± standard deviation (SD) from three independent measurements (n = 3). The differences in the concentrations of the analyzed secondary plant metabolites were compared using the ANOVA method (one-factor or two-factor with repetitions). The differences in the amounts of individual compounds were considered statistically significant when the determined *p*-value was lower than 0.05 and the Fisher coefficient (*F*) value was higher than the tabulated critical value (*Fcrit*).

## Figures and Tables

**Table 1:** Structures of positional isomers of mono- and di-caffeoylquinic acids; quinic acid (Q), caffeic acid (CA).

No.	Compound Name	Abbreviation	R_1_	R_3_	R_4_	R_5_
1	1-*O*-caffeoylquinic acid	1-CQA	CA	H	H	H
2	3-*O*-caffeoylquinic acid (neochlorogenic acid)	3-CQA	H	CA	H	H
3	5-*O*-caffeoylquinic acid (chlorogenic acid)	5-CQA	H	H	H	CA
4	4-*O*-caffeoylquinic acid (chlorogenic acid)	4-CQA	H	H	CA	H
5	1,3-di-*O*-caffeoylquinic acid	1,3-di-CQA	CA	CA	H	H
6	1,4-di-*O*-caffeoylquinic acid	1,4-di-CQA	CA	H	CA	H
7	1,5-di-*O*-caffeoylquinic acid	1,5-di-CQA	CA	H	H	CA
8	3,4-di-*O*-caffeoylquinic acid	3,4-di-CQA	H	CA	CA	H
9	3,5-di-*O*-caffeoylquinic acid	3,5-di-CQA	H	CA	H	CA
10	4,5-di-*O*-caffeoylquinic acid	4,5-di-CQA	H	H	CA	CA

**Table 2:** Data used to identify 5-CQA and 1,3-diCQA degradation/transformation products (compound numbering consistent with peak numbering in Figure 2).

Compound Number	*t_R_* [min]	λ_max_ [nm]	MS^1^ Parent Ion [M-H]^−^ *m*/*z*	MS^2^ Characteristic Ions (Intensity, %) *m*/*z* (%)	Compound Shortcut
1	13.1	300*, 326	353	191 (100), 179 (5)	trans-5-CQA (5-CQA)
2	16.2	300*, 322	515	353	1,3-diCQA
3	8.5	300*, 325	353	191 (100)	1-CQA
4	9.9	300*, 328	353	191 (100), 179 (91), 173 (1);135 (14)	3-CQA
5	13.7	300*, 328	353	179 (100), 191 (15), 173 (88), 135 (9)	4-CQA
6	16.3	300, 315	353	191 (100), 179 (2)	cis-5-CQA
7	17.1	300*, 321	179	135 (100)	CA
8	17.3	280	194	161 (100)	CME
9	23.9	300*, 323	515	353	3,4-diCQA
10	24.6	300*, 328	515	353	1,5-diCQA
11	25.3	300, 323	515	353	3,5-diCQA
12	25.7	300*, 320	515	353	cis-1,5-diCQA
13	27.0	300*, 328	515	353	4,5-diCQA
14	21.2	300*, 323	515	353	1,4-diCQA

**Table 3:** The amounts of parent compounds remaining after their simulated extraction by MASE, UASE, PLE and SSDM techniques of solutions containing 10 mg of each standard [mg ± SD].

Compound Shortcut	MASE			UASE			PLE			SSDM
	50% 30 min	100% 30 min	100% 60 min	37 kHz 30 min	80 kHz 30 min	37 kHz 60 min	50 °C	100 °C	150 °C	
5-CQA	6.89 ± 0.28	4.75 ± 0.18	4.05 ± 0.24	6.03 ± 0.23	7.83 ± 0.12	4.60 ± 0.14	9.64 ± 0.49	8.12 ± 0.29	4.45 ± 0.23	9.79 ± 0.13
1,3-diCQA	4.24 ± 0.21	3.53 ± 0.18	3.24 ± 0.07	3.95 ± 0.22	5.32 ± 0.18	3.22 ± 0.07	4.48 ± 0.45	3.92 ± 0.28	3.68 ± 0.07	9.98 ± 0.49

**Table 4:** Statistical analysis of the influence of assisted extraction techniques and their characteristic variables/conditions on the estimations of 5-CQA and 1,3-diCQA quantities after simulated extraction process (F_crit_ = 7708).

Compound Shortcut	MASE/SSDM			UASE/SSDM			PLE/SSDM		
	50%_30 min	100%_30 min	100%_60 min	37 kHz_30 min	80 kHz_30 min	37 kHz_60 min	50 °C	100 °C	150 °C
	F-Value (*p*)	F-Value (*p*)	F-Value (*p*)	F-Value (*p*)	F-Value (*p*)	F-Value (*p*)	F-Value (*p*)	F-Value (*p*)	F-Value (*p*)
5-CQA	270.75 (8.00 × 10^−5^)	1587.16 (2.37 × 10^−6^)	1345.38 (3.30 × 10^−6^)	614.71 (1.57 × 10^−2^)	386.98 (3.94 × 10^−5^)	2259.23 (1.17 × 10^−6^)	0.250 (0.644)	125.06 (3.64 × 10^−4^)	857.20 (8.08 × 10^−6^)
1,3-diCQA	347.23 (4.88 × 10^−5^)	461.42 (2.78 × 10^−5^)	561.08 (1.88 × 10^−5^)	384.30 (4.00 × 10^−5^)	241.12 (1.00 × 10^−4^)	563.69 (1.87 × 10^−6^)	206.00 (1.37 × 10^−4^)	348.27 (4.85 × 10^−5^)	489.81 (2.47 × 10^−5^)

**Table 5:** Basic characteristics of calibration curves.

Compound Shortcut	Slope (a)	Intercept (b)	R^2^	Slope Estimation Error	Intercept Estimation Error	Calibration Curve Error
5-CQA	2464.72	−0.3093	0.9999	15.4832	0.34853	0.09853
1,3-di-CQA	3077.74	1.0663	0.9997	113.546	0.82738	0.67254
1,5-di-CQA	2560.02	0.2908	0.9996	89.5472	0.89751	0.86738
CA	0.22433	−0.0080	0.9958	0.01282	0.04966	0.01959

## Data Availability

Data are contained within the article.

## Supplementary Materials

The following supporting information can be downloaded at: https://www.mdpi.com/article/10.3390/molecules29245861/s1, Figure S1: Representative chromatograms of 5-CQA (left part of the figure: A, C, E) and 1,3-diCQA (right part of the figure: B, D, F) standard solutions after simulated UASE extraction performed at 37 kHz and 80 kHz for 30 min and/or 60 min of exposure to ultrasound: 5-CQA (peak 1), 1,3-diCQA (2), 1-CQA (3), 3-CQA (4), 4-CQA (5), *cis*-5-CQA (6), CA (7), CME (8), 1,5-diCQA (10), *cis*-1,5-diCQA (12), 4,5-diCQA (13), 1,4-diCQA (14); Figure S2: Representative chromatograms of 5-CQA (left part of the figure: A, C, E) and 1,3-diCQA (right part of the figure: B, D, F) standard solutions after simulated PLE extraction; Figure S3: Exemplary chromatograms of 1,3-diCQA standard solutions subjected to simulated PLE extraction using water as an extractant and temperature of 100 °C for 10 min (A) and 20 min (B) (40 bar); Figure S4: System chromatograms, for data from Figure 2, of 5-CQA (A) and 1,3-diCQA (B) standard solutions without and after simulated MASE extraction at 50 and 100% generator power for 30 and 60 min, recorded at 330 nm (detailed description in the text); Figure S5: System chromatograms, for data from Figure 6, of extracts of coltsfoot (A), yarrow (B), chamomile (C), artichoke leaves (D), green coffee beans (E), artichoke bud “heart” (F) and tansy herb (G) obtained by UASE at 37 kHz; Table S1: ANOVA results assessing the impact of MASE generator power and time on the amounts of mono-CQAs estimated in the *trans*-5-CQA standard solution after the simulated extraction process (F_crit_ = 7.708); Table S2: ANOVA results assessing the impact of MASE generator power and time on the amounts of mono- and diCQAs estimated in the 1,3-diCQA standard solution after the simulated extraction process (Fcrit = 7.708); Table S3: ANOVA results assessing the influence of UASE process frequency and time on the amounts of mono-CQAs estimated in the *trans*-5-CQA standard solution after the simulated extraction process (Fcrit = 7.708); Table S4: ANOVA results assessing the influence of UASE process frequency and time on the amounts of mono- and diCQAs estimated in the 1,3-diCQA standard solution after the simulated extraction process (Fcrit = 7.708); Table S5: ANOVA results assessing the influence of the PLE process temperature on the amounts of CQAs estimated in the 5-CQA standard solution after the simulated extraction process (Fcrit = 5.143); Table S6: ANOVA results assessing the influence of the PLE process temperature on the amounts of CQAs estimated in the 1,3-diCQA standard solution after the simulated extraction process (Fcrit = 5.143); Table S7: Quantity of chlorogenic acids and their derivatives estimated in green coffee beans extracts obtained by assisted extraction techniques and SSDM (expressed in µg/g ± SD); Table S8: Statistical analysis of the effect of assisted extraction techniques and their characteristic variables/conditions on the assessment of the quantity of chlorogenic acids in green coffee beans extracts (Fcrit = 7.708); Table S9: Quantity of chlorogenic acids and their derivatives estimated in artichoke (*Cynara cardunculus var scolymus* L.) leaf extracts obtained by assisted extraction techniques and SSDM (expressed in µg/g ± SD); Table S10: Statistical analysis of the effect of assisted extraction techniques and their characteristic variables/conditions on the assessment of the quantity of chlorogenic acids in artichoke leaf extracts (Fcrit = 7.708); Table S11: Quantity of chlorogenic acids and their derivatives estimated in artichoke (*Cynara cardunculus var scolymus* L.) heart extracts obtained using assisted extraction techniques and SSDM (expressed in µg/g ± SD); Table S12: Statistical analysis of the effect of assisted extraction techniques and their characteristic variables/conditions on the assessment of the quantity of chlorogenic acids in artichoke heart extracts (Fcrit = 7.708); Table S13: Quantity of chlorogenic acids and their derivatives estimated in coltsfoot (*Tussilago farfara* L.) herb extracts obtained using assisted extraction techniques and SSDM (expressed in µg/g ± SD); Table S14: Statistical analysis of the effect of assisted extraction techniques and their characteristic variables/conditions on the assessment of the quantity of chlorogenic acids in coltsfoot extracts (Fcrit = 7.708); Table S15: Quantity of chlorogenic acids and their derivatives estimated in chamomile (*Matricaria* L.) flower extracts obtained by assisted extraction techniques and SSDM (expressed in µg/g ± SD); Table S16: Statistical analysis of the effect of assisted extraction techniques and their characteristic variables/conditions on the assessment of the quantity of chlorogenic acids in chamomile extracts (Fcrit = 7.708); Table S17: Quantity of chlorogenic acids and their derivatives estimated in tansy (*Tanacetum* L.) flower extracts obtained by assisted extraction techniques and SSDM (expressed in µg/g ± SD); Table S18: Statistical analysis of the effect of assisted extraction techniques and their characteristic variables/conditions on the assessment of the quantity of chlorogenic acids in tansy extracts (Fcrit = 7.708); Table S19: Quantity of chlorogenic acids and their derivatives estimated in yarrow (*Achillea* L.) flower extracts obtained using assisted extraction techniques and SSDM (expressed in µg/g ± SD); Table S20: Statistical analysis of the effect of assisted extraction techniques and their characteristic variables/conditions on the assessment of the quantity of chlorogenic acids in yarrow extracts (Fcrit = 7.708).

## References

[1] Reshi Z.A., Ahmad W., Lukatkin A.S., Javed S.B. (2023). From Nature to Lab: A Review of Secondary Metabolite Biosynthetic Pathways, Environmental Influences, and In Vitro Approaches. Metabolites.

[2] Crozier A., Yokota T., Jaganath I.B., Marks S., Saltmarsh M., Clifford M.N. (2007). Secondary Metabolites in Fruits, Vegetables, Beverages and Other Plant-based Dietary Components. Plant Secondary Metabolites.

[3] Choi Y.E., Choi S.I., Han X., Men X., Jang G.W., Kwon H.Y., Kang S.R., Han J.S., Lee O.A. (2020). Radical Scavenging-Linked Anti-Adipogenic Activity of *Aster scaber* Ethanolic Extract and Its Bioactive Compound. Antioxidants.

[4] Williamson G. (2020). Protection against developing type 2 diabetes by coffee consumption: Assessment of the role of chlorogenic acid and metabolites on glycaemic responses. Food Funct..

[5] Kusumah J., Gonzalez de Mejia E. (2022). Coffee constituents with antiadipogenic and antidiabetic potentials: A narrative review. Food Chem. Toxicol..

[6] Ludwig I.A., Clifford M.N., Lean M.E.J., Ashihara H., Crozier A. (2014). Coffee: Biochemistry and potential impact on health. Food Funct..

[7] Wianowska D., Gil M. (2019). Recent advances in extraction and analysis procedures of natural chlorogenic acids. Phytochem. Rev..

[8] Jaiswal R., Kiprotich J., Kuhnert N. (2011). Determination of the hydroxycinnamate profile of 12 members of the Asteraceae family. Phytochemistry.

[9] Wu J., Qian Y., Mao P., Chen L., Lu Y., Wang H. (2013). Separation and identification of phenolic compounds in canned artichoke by LC/DAD/ESI-MS using core-shell C18 column: A comparative study. J. Chromatogr. B Biomed. Appl..

[10] Yang M., Ma Y., Wang Z., Khan A., Zhou W., Zhao T., Cao J., Cheng G., Cai S. (2020). Phenolic constituents, antioxidant and cytoprotective activities of crude extract and fractions from cultivated artichoke inflorescence. Ind. Crops Prod..

[11] Tena M.T., Martínez-Moral M.P., Cardozo P.W. (2015). Determination of caffeoylquinic acids in feed and related products by focused ultrasound solid-liquid extraction and ultra-high performance liquid chromatography-mass spectrometry. J. Chromatogr. A.

[12] Haddouchi F., Chaouche T.M., Ksouri R., Larbat R. (2021). Leafy Stems of *Phagnalon saxatile* subsp. *Saxatile* from Algeriaas a Source of Chlorogenic Acids and Flavonoids with Antioxidant Activity: Characterization and Quantification Using UPLC-DAD-ESI-MS^n^. Metabolites.

[13] Jaiswal R., Müller H., Müller A., Karar M.G.E., Kuhnert N. (2014). Identification and characterization of chlorogenic acids, chlorogenic acid glycosides and flavonoids from Lonicera henryi L. (*Caprifoliaceae*) leaves by LC-MSn. Phytochemistry.

[14] Gouveia S.C., Castilho P.C. (2012). Validation of a HPLC-DAD-ESI/MS n method for caffeoylquinic acids separation, quantification and identification in medicinal Helichrysum species from Macaronesia. Food Res. Int..

[15] Dias M.I., Barros L., Dueñas M., Pereira E., Carvalho A.M., Alves R.C., Oliveira M.B.P.P., Santos-Buelga C., Ferreira I.C.F.R. (2013). Chemical composition of wild and commercial Achillea millefolium L. and bioactivity of the methanolic extract, infusion and decoction. Food Chem..

[16] Wianowska D., Typek R., Dawidowicz A.L. (2015). How to eliminate the formation of chlorogenic acids artefacts during plants analysis? Sea sand disruption method (SSDM) in the HPLC analysis of chlorogenic acids and their native derivatives in plants. Phytochemistry.

[17] Wianowska D., Typek R., Dawidowicz A.L. (2015). Chlorogenic acid stability in pressurized liquid extraction conditions. J. AOAC Int..

[18] Dawidowicz A.L., Typek R. (2017). Transformation of chlorogenic acids during the coffee beans roasting process. Eur. Food Res. Technol..

[19] Wianowska D., Dawidowicz A.L., Bernacik K., Typek R. (2017). Determining the true content of quercetin and its derivatives in plants employing SSDM and LC–MS analysis. Eur. Food Res. Technol..

[20] Deshpande S., Jaiswal R., Matei M.F., Kuhnert N. (2014). Investigation of acyl migration in mono- and dicaffeoylquinic acids under aqueous basic, aqueous acidic, and dry roasting conditions. J. Agric. Food Chem..

[21] Yang J., Yao L., Gong K., Li K., Sun L., Cai W. (2022). Identification and Quantification of Chlorogenic Acids from the Root Bark of *Acanthopanax gracilistylus* by UHPLC-Q-Exactive Orbitrap Mass Spectrometry. ACS Omega.

[22] Ramabulana A.T., Steenkamp P., Madala N., Duber I.A. (2020). Profiling of Chlorogenic Acids from *Bidens pilosa* and Differentiation of Closely Related Positional Isomers with the Aid of UHPLC-QTOF-MS/MS-Based In-Source Collision-Induced Dissociation. Metabolites.

[23] Pérez R., Burgos V., Marín V., Camins A., Olloquequi J., González-Chavarría I., Ulrich H., Wyneken U., Luarte A., Ortiz L. (2023). Caffeic Acid Phenethyl Ester (CAPE): Biosynthesis, Derivatives and Formulations with Neuroprotective Activities. Antioxidants.

[24] Clifford M.N., Kirkpatrick J., Kuhnert N., Roozendaal H., Salgado P.R. (2008). LC-MSn analysis of the cis isomers of chlorogenic acids. Food Chem..

[25] Cangeloni L., Bonechi C., Leone G., Consumi M., Andreassi M., Magnani A., Rossi C., Tamasi G. (2022). Characterization of Extracts of Coffee Leaves (*Coffea arabica* L.) by Spectroscopic and Chromatographic/Spectrometric Techniques. Foods.

[26] Cirkva V., Relich S. (2010). Microwave Photochemistry and Photocatalysis. Part 1: Principles and Overview. Curr. Org. Chem..

