# Supplementary material for: Stability of Selected Phenolic Acids Under Simulated and Real Extraction Conditions from Plants
Source: Molecules. 2024 Dec 12;29(24):5861. doi: 10.3390/molecules29245861 (PMC11677208; doi:10.3390/molecules29245861)
Supplement: Supplementary file 1 [file molecules-29-05861-s001.zip › molecules-3317565-supplementary.pdf]

2.2 Assessment of the amount of 5-CQA and 1,3-diCQA and their derivatives under simulated MASE conditions

Table S1. ANOVA results assessing the impact of MASE generator power and time on the amount of mono-CQAs estimated in the *trans*-5-CQA standard solution after the simulated extraction process (F_{crit} = 7.708).

**Compound**	**Effect of generator power**		**Effect of time**	
**shortcut**	***F***	***p***	***F***	***p***
1-CQA	-	-	42.416	0.003
3-CQA	11.249	0.028	2.013	0.229
**5-CQA**	**196.138**	**0.000**	**22.213**	**0.009**
4-CQA	0.553	0.498	33.153	0.004
cis-5-CQA	8.890	0.041	34.531	0.004

Table S2. ANOVA results assessing the impact of MASE generator power and time on the amount of mono- and diCQAs estimated in the 1,3-diCQA standard solution after the simulated extraction process (Fcrit = 7.708).

**Compound**	**Effect of generator power**		**Effect of time**	
**shortcut**	***F***	***p***	***F***	***p***
3-CQA	-	-	-	-
5-CQA	1.036	0.366	0.102	0.765
4-CQA	2.303	0.204	0.266	0.633
1,3-diCQA	**19.411**	**0.012**	**6.810 (a)**	**0.059**
CA	40.881	0.003	47.372	0.002
CME	45.876	0.002	63.104	0.001
1,4-diCQA	-	-	-	-
3,4-diCQA	0.440	0.543	0.038	0.854
1,5-diCQA	17.328	0.014	1.005	0.378
3,5-diCQA	2.194	0.212	9.912	0.0345
4,5-diCQA	0.487	0.523	0.282	0.623

2.3 Stability/quantity studies of 5-CQA and 1,3-diCQA and their derivatives under simulated UASE extraction conditions

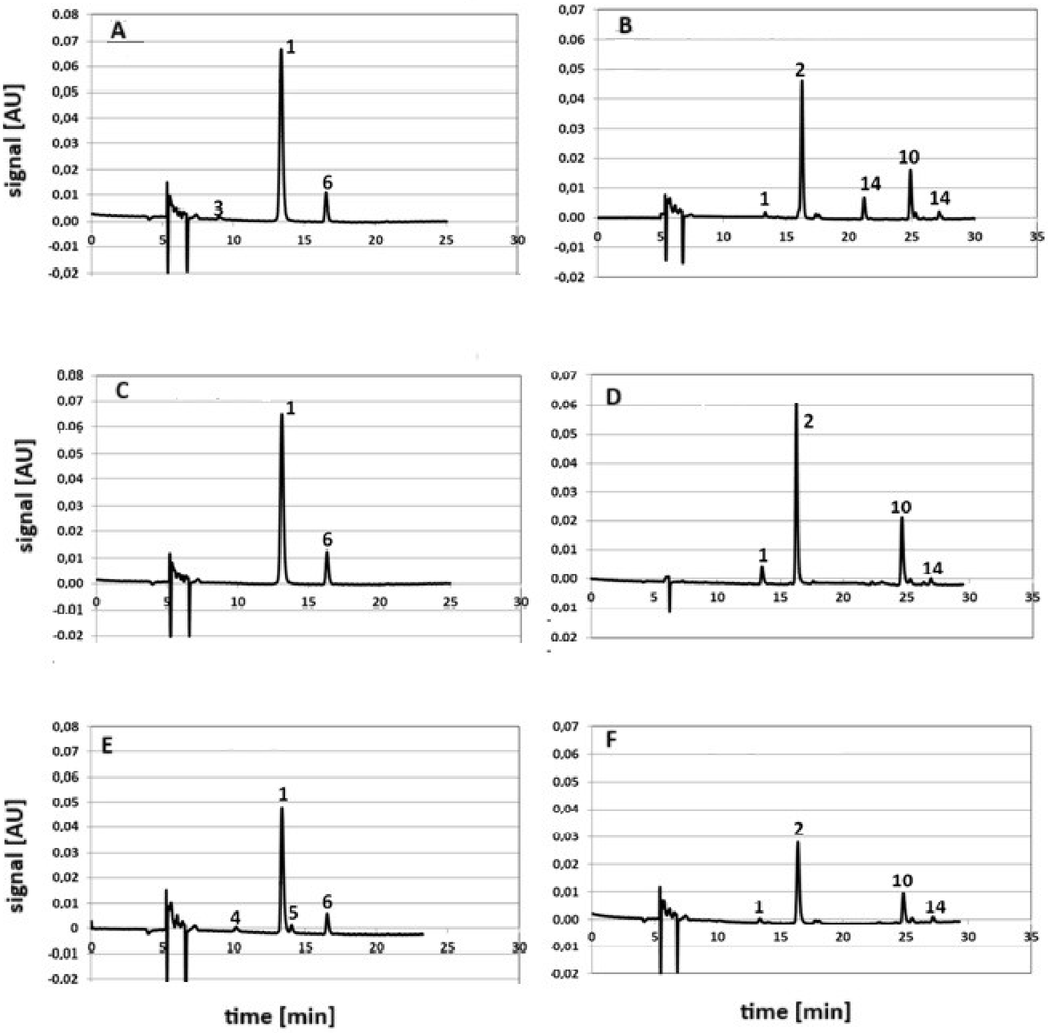

Figure S1. Representative chromatograms of 5-CQA (left part of the figure: A, C, E) and 1,3-diCQA (right part of the figure: B, D, F) standard solutions after simulated UASE extraction performed at 37kHz and 80kHz for 30min and/or 60min of exposure to ultrasounds (detailed description in the text).

Table S3. ANOVA results assessing the influence of UASE process frequency and time on the amount of mono-CQAs estimated in the *trans*-5-CQA standard solution after the simulated extraction process (Fcrit = 7.708).

**Compound shortcut**	**Effect of frequency** *F*	*p*	**Effect of time** *F*	*p*
1-CQA	20.291	0.011	-	-
3-CQA	-	-	24.814	0.008
5-CQA	141.131	0.000	85.569	0.000
4-CQA	-	-	30.0205	0.005
cis-5-CQA	9.246	0.038	42.471	0.003

Table S4. ANOVA results assessing the influence of UASE process frequency and time on the amount of mono- and diCQAs estimated in the 1,3-diCQA standard solution after the simulated extraction process (Fcrit = 7.708).

Compound shortcut	Effect of frequency *F*	*p*	Effect of time *F*	*p*
3-CQA	-	-	-	-
5-CQA	22.200	0.010	2.100	0.221
4-CQA	-	-	-	-
1,3-diCQA	71.830	0.002	30.544	0.005
CA	0.274	0.628	0.176	0.696
CME	8.730	0.042	0.0277	0.876
1,4-diCQA	92.774	0.001	-	-
3,4-diCQA	-	-	19.005	0.012
1,5-diCQA	9.959	0.034	39.341	0.003
3,5-diCQA	0.008	0.931	1.392	0.303
4,5-diCQA	0.223	0.662	0.261	0.636

2.4 Studies on the stability/quantity of 5-CQA and 1,3-diCQA and their derivatives in simulated PLE extraction conditions

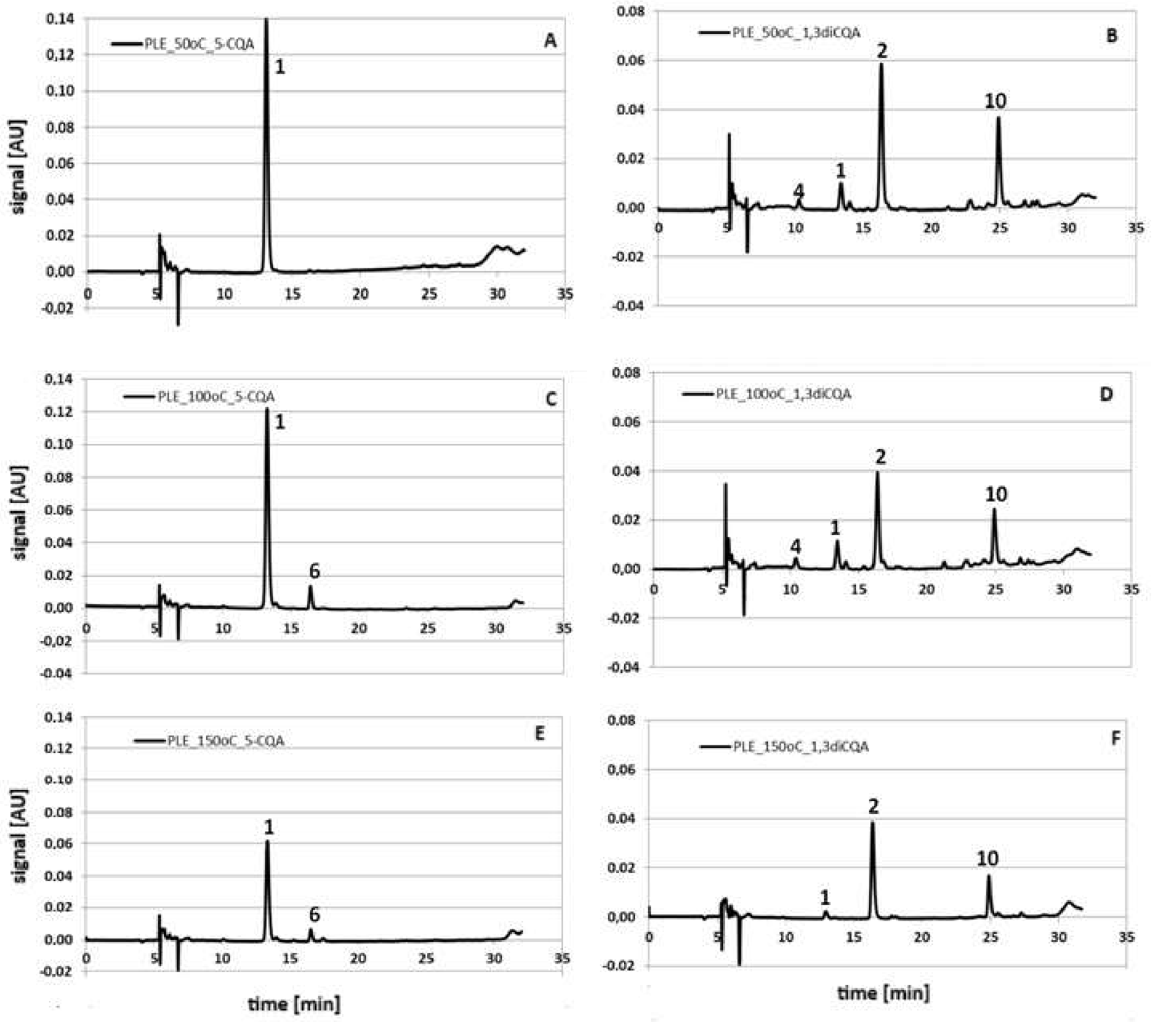

Figure S2. Representative chromatograms of 5-CQA (left part of the figure: A, C, E) and 1,3-diCQA (right part of the figure: B, D, F) standard solutions after simulated PLE extraction.

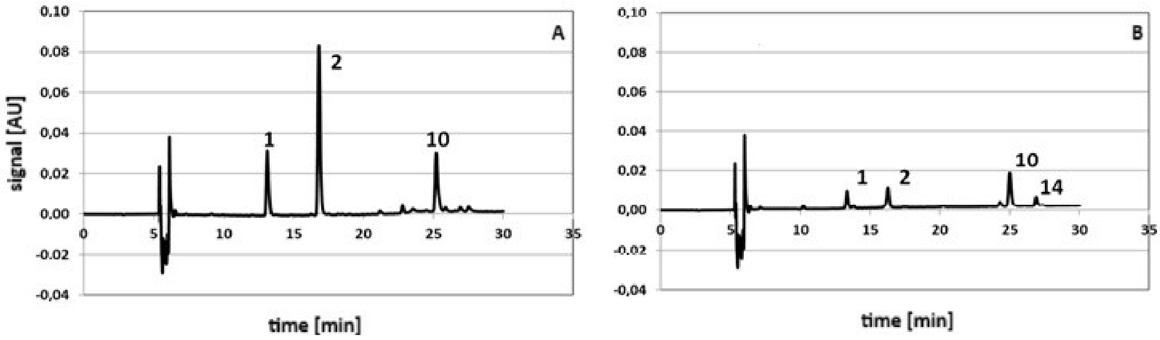

Figure S3. Exemplary chromatograms of 1,3-diCQA standard solutions subjected to simulated PLE extraction using water as an extractant and temperature of 100°C for 10min (A) and 20min (B) (40 bar).

Table S5. ANOVA results assessing the influence of the PLE process temperature on the amount of CQAs estimated in the 5-CQA standard solution after the simulated extraction process (Fcrit = 5.143).

**Compound shortcut**	**Effect of temperature**	
	F *	*p*
3-CQA	2.195	0.192
5-CQA	226.217	0.000
4-CQA	0.997	0.422
cis-5-CQA	75.888	0.000

Table S6. ANOVA results assessing the influence of the PLE process temperature on the amount of CQAs estimated in the 1,3-diCQA**s**tandard solution after the simulated extraction process (Fcrit = 5.143).

**Compound shortcut**	**Effect of temperature**	
	F *	*p*
3-CQA	12.300	0.008
5-CQA	16.463	0.004
4-CQA	36.531	0.000
1,3-diCQA	5.278	0.048
1,4-diCQA	12.078	0.008
3,4-diCQA	3.818	0.085
1,5-diCQA	41.602	0.000
3,5-diCQA	6.378	0.033
4,5-diCQA	18.633	0.003

Table S7. Quantity of chlorogenic acids and their derivatives estimated in green coffee beans extracts obtained by assisted extraction techniques and SSDM (expressed in µg/g ± SD).

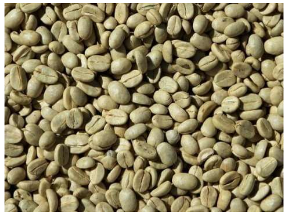

Compound shortcut	UASE 37 kHz	UASE 80 kHz	MASE 100%	PLE 50°C	PLE 100°C	PLE 150°C	SSDM
1-CQA	-	-	-	-	-	-	-
3-CQA	4112 ± 143	3918 ± 352	4230 ± 286	3953 ± 158	4175 ± 285	**5062 ± 143**	4213 ± 241
5-CQA	50632 ±1959	46955 ± 3085	53747 ± 2148	47938 ± 1886	49249 ± 2556	52007 ± 1383	48960 ± 2248
4-CQA	6028 ±457	5088 ± 758	**7089 ± 456**	5903 ± 573	**6503 ± 268**	**7831 ± 667**	5413 ± 376
cis-5-CQA	**549 ±18**	**438 ± 26**	**847 ± 46**	655 ± 52	**488 ± 33**	**427 ± 15**	722 ± 39
1,3-diCQA	-	-	-	-	-	-	-
CA	**428 ± 22**	**355 ± 25**	**567 ± 29**	**147 ± 13**	254 ± 18	**98 ± 8**	249 ± 13
CME	-	-	**-**	-	-	-	-
1,4-diCQA	-	-	**-**	-	-	-	-
3,4-diCQA	1979 ± 105	1789 ± 115	**2265 ± 100**	1774 ± 89	1886 ± 97	**242 ± 126**	1878 ± 89
1,5-diCQA	4770 ± 288	4560 ± 239	**5779 ± 228**	4491 ± 180	**4251 ± 180**	4879 ±256	4950 ± 287
3,5-diCQA	-	-	**-**	-	-	-	-
4,5-diCQA	3066 ± 155	2880 ± 196	**4000 ± 225**	**2609 ± 187**	3290 ± 187	**4106 ± 215**	3032 ± 168
∑	**71564**	**65984**	**79524**	**67470**	**75019**	**76831**	**64519**

Table S8. Statistical analysis of the effect of assisted extraction techniques and their characteristic variables/conditions on the assessment of the quantity of chlorogenic acids in green coffee beans extracts (Fcrit = 7.708).

**Compound shortcut**	**UASE/SSDM**				**MASE/SSDM**				**PLE/SSDM**			
	**37 kHz**		**80 kHz**		**100%**		**50°C**		**100°C**		**150°C**	
	F	***p***	***F***	***p***	***F***	***p***	***F***	***p***	***F***	***p***	***F***	***p***
1-CQA	-	-	-	-	-	-	-	-	-	-	-	-
3-CQA	0.40	$5\cdot10^{-01}$	1.43	$3\cdot10^{-01}$	0.01	$9\cdot10^{-02}$	2.44	$2\cdot10^{-01}$	0.03	$9\cdot10^{-01}$	**27.53**	$6\cdot10^{-03}$
5-CQA	0.94	$4\cdot10^{-01}$	0.83	$4\cdot10^{-01}$	7.11	$5\cdot10^{-03}$	0.36	$6\cdot10^{-01}$	0.02	$9\cdot10^{-01}$	4.00	$1\cdot10^{-01}$
4-CQA	3.24	$1\cdot10^{-01}$	0.44	$5\cdot10^{-01}$	**24.12**	$8\cdot10^{-03}$	1.53	$3\cdot10^{-01}$	**16.73**	$1\cdot10^{-03}$	**29.93**	$5\cdot10^{-03}$
cis-5-CQA	**49.10**	$2\cdot10^{-03}$	**111.8**	$4\cdot10^{-04}$	**13.15**	$2\cdot10^{-03}$	3.18	$1\cdot10^{-01}$	**63.10**	$1\cdot10^{-03}$	**150.44**	$3\cdot10^{-04}$
1,3-diCQA	-	-	**-**	-	**-**	-	-	-	-	-	-	-
CA	**142.19**	$3\cdot10^{-04}$	**41.05**	$3\cdot10^{-03}$	**305.10**	$6\cdot10^{-05}$	**92.70**	$7\cdot10^{-05}$	0.14	$7\cdot10^{-01}$	**287.40**	$7\cdot10^{-05}$
CME		-	-			-			-	-	-	-
1,4-diCQA												
3,4-diCQA	1.63	$2\cdot10^{-01}$	1.09	$3\cdot10^{-01}$	**25.13**	$7\cdot10^{-03}$	2.0	$2\cdot10^{-01}$	0.11	$9\cdot10^{-01}$	**37.00**	$2\cdot10^{-03}$
1,5-diCQA	0.58	$5\cdot10^{-01}$	3.26	$1\cdot10^{-01}$	**15.3**	$2\cdot10^{-02}$	3.98	$1\cdot10^{-01}$	**12.73**	$2\cdot10^{-02}$	0.10	$8\cdot10^{-01}$
3,5-diCQA												
4,5-diCQA	0.07	$8\cdot10^{-01}$	1.04	$4\cdot10^{-01}$	**35.78**	$4\cdot10^{-03}$	**10.20**	$3\cdot10^{-02}$	3.18	$2\cdot10^{-01}$	**46.44**	$2\cdot10^{-03}$

Table S9. Quantity of chlorogenic acids and their derivatives estimated in artichoke (*Cynara cardunculus var scolymus*L.) leaf extracts obtained by assisted extraction techniques and SSDM (expressed in μg/g ± SD).

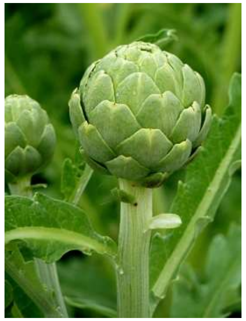

Compound shortcut	UASE 37 kHz	UASE 80 kHz	MASE 100%	PLE 50oC	PLE 100oC	PLE 150oC	SSDM
1-CQA	467 ± 27	479 ± 42	482 ± 28	515 ± 29	511 ± 29	**645 ± 36**	475 ± 17
3-CQA	266 ± 16	232 ± 12	249 ± 15	266 ± 21	247 ± 15	**340 ± 20**	236 ± 13
5-CQA	8198 ± 452	7675 ± 376	8253 ± 505	**8890 ± 536**	**8858 ± 435**	**9976 ± 491**	7704 ± 377
4-CQA	342 ± 23	328 ± 32	**402 ± 42**	**384 ± 35**	**368 ± 24**	**595 ± 36**	296 ± 21
cis-5-CQA	**89 ± 6**	75 ± 10	**116 ± 8**	56 ±13	55 ± 5	**127 ± 8**	66 ± 5
1,3-diCQA	1243 ± 72	**1634 ± 92**	**1771 ± 99**	**1037 ± 62**	**1095 ± 85**	**1198 ± 70**	1409 ±80
CA	**125 ± 9**	**165 ± 11**	**185 ± 12**	**76 ± 7**	**297 ± 18**	98 ± 8	102 ± 8
CME	-	-	-	-	-	-	-
1,4-diCQA	**2111 ± 101**	**2136 ± 122**	1804 ± 85	**1444 ± 124**	**1083 ± 135**	**1416 ± 66**	1769 ± 99
3,4-diCQA	**368 ± 22**	339 ± 21	**1074 ± 58**	**257 ± 17**	280 ± 18	334 ± 21	316 ± 20
1,5-diCQA*<br>3,5-diCQA*	5825 ± 281	5298 ± 415	5189 ± 249	**6424 ± 355**	**5987 ± 289**	**6297 ± 305**	5341 ± 257
4,5-diCQA	***1602 ± 89***	1048 ± 61	1027 ± 60	**826 ± 50**	1130 ± 50	969 ± 57	1007 ± 59
∑	**20636**	**19411**	**20551**	**20174**	**19613**	**21997**	**18618**

*inseparablepeaks

Table S10. Statistical analysis of the effect of assisted extraction techniques and their characteristic variables/conditions on the assessment of the quantity of chlorogenic acids in artichoke leaf extracts (Fcrit = 7.708).

**Compound shortcut**	**UASE/SSDM**				**MASE/SSDM**				**PLE/SSM**			
	**37 kHz**		**80 kHz**		**100%**		**50°C**		**100°C**		**150°C**	
	F	***p***	***F***	***p***	***F***	***p***	***F***	***p***	***F***	***p***	***F***	***p***
1-CQA	0.16	$7\cdot10^{-01}$	0.02	$9\cdot10^{-01}$	0.13	$7\cdot10^{-01}$	4.16	$1\cdot10^{-01}$	3.54	$1\cdot10^{-01}$	**55.5**	$2\cdot10^{-03}$
3-CQA	6.14	$6\cdot10^{-02}$	0.11	$8\cdot10^{-01}$	1.36	$3\cdot10^{-01}$	4.41	$1\cdot10^{-01}$	0.86	$4\cdot10^{-01}$	**57.72**	$2\cdot10^{-03}$
5-CQA	2.11	$2\cdot10^{-01}$	0.01	$9\cdot10^{-01}$	2.27	$2\cdot10^{-01}$	**9.82**	$4\cdot10^{-02}$	**12.04**	$3\cdot10^{-02}$	**40.39**	$3\cdot10^{-03}$
4-CQA	6.45	$6\cdot10^{-02}$	2.08	$2\cdot10^{-02}$	**15.11**	$2\cdot10^{-02}$	**13.74**	$2\cdot10^{-02}$	**14.84**	$2\cdot10^{-02}$	**156.50**	$2\cdot10^{-04}$
cis-5-CQA	**23.92**	$8\cdot10^{-03}$	2.11	$2\cdot10^{-01}$	**84.32**	$8\cdot10^{-04}$	1.52	$3\cdot10^{-01}$	7.47	$5\cdot10^{-02}$	**115.83**	$4\cdot10^{-04}$
1,3-diCQA	7.05	$6\cdot10^{-02}$	**10.21**	$3\cdot10^{-02}$	**24.34**	$8\cdot10^{-03}$	**40.24**	$3\cdot10^{-03}$	**21.56**	$9\cdot10^{-03}$	**11.77**	$3\cdot10^{-02}$
CA	**9.71**	$3\cdot10^{-02}$	**61.54**	$1\cdot10^{-03}$	**93.84**	$6\cdot10^{-04}$	**19.35**	$1\cdot10^{-02}$	**294.32**	$7\cdot10^{-05}$	0.38	$5\cdot10^{-01}$
CME	-	-	-	-	-	-	-	-	-	-	-	-
1,4-diCQA	**17.58**	$1\cdot10^{-02}$	**16.37**	$2\cdot10^{-02}$	0.21	$7\cdot10^{-01}$	**12.60**	$2\cdot10^{-02}$	**50.32**	$2\cdot10^{-03}$	**26.41**	$7\cdot10^{-04}$
3,4-diCQA	**9.40**	$4\cdot10^{-02}$	2.02	$2\cdot10^{-01}$	**463.80**	$3\cdot10^{-05}$	**15.17**	$2\cdot10^{-02}$	5.41	$8\cdot10^{-02}$	1.30	$3\cdot10^{-01}$
1,5-diCQA* 3,5-diCQA*	4.84	$9\cdot10^{-02}$	0.02	$9\cdot10^{-01}$	0.54	$5\cdot10^{-01}$	**18.33**	$1\cdot10^{-02}$	**8.35**	$4\cdot10^{-02}$	**17.24**	$1\cdot10^{-0}$
4,5-diCQA	**92.66**	$7\cdot10^{-04}$	0.68	$4\cdot10^{-01}$	0.16	$7\cdot10^{-03}$	**16.14**	$2\cdot10^{-02}$	7.51	$5\cdot10^{-02}$	0.63	$5\cdot10^{-01}$

*inseparablepeaks

Table S11. Quantity of chlorogenic acids and their derivatives estimated in artichoke (*Cynara cardunculus var scolymus*L.) heart extracts obtained using assisted extraction techniques and SSDM (expressed in µg/g ± SD).

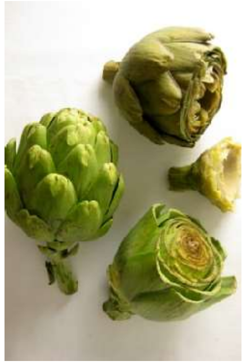

**Compound shortcut**	**UASE** 37 kHz	**UASE** 80 kHz	**MASE** 100%	**PLE** 50°C	**PLE** 100°C	**PLE** 150°C	**SSDM**
1-CQA	-	-	-	-	-	-	-
3-CQA	41172 ± 209	3870 ± 126	**4550 ± 231**	3919 ± 214	4381 ± 222	**5225 ± 264**	3980 ± 241
5-CQA	**28750 ±452**	29425 ± 1463	**32036 ± 505**	**30144 ± 536**	**30745 ± 1529**	**32074 ± 1596**	26318 ± 1308
4-CQA	**4549 ±233**	**4708 ± 328**	6017 ± 426	6038 ± 352	6740 ± 343	**7474 ± 380**	6360 ± 324
cis-5-CQA	368 ±20	346 ± 10	**324 ± 18**	**294 ± 13**	**252 ± 15**	**169 ± 10**	372 ± 21
1,3-diCQA	3743 ± 197	4076 ± 214	3588 ± 189	**3249 ± 172**	4102 ± 85	3803 ± 200	3916 ± 206
CA	-	-	-	-	-	-	-
CME	-	-	-	-	-	-	-
1,4-diCQA	-	-	-	-	-	-	-
3,4-diCQA	2887 ± 148	2658 ± 137	**3375 ± 173**	2588 ± 133	2750 ± 142	3051 ± 157	2724 ± 140
1,5-diCQA	**14545 ± 717**	13704 ± 415	**9631 ± 472**	11795 ± 355	12383 ± 609	**14147 ± 697**	12569 ± 618
3,5-diCQA	**-**	-	**-**	-	-	-	-
4,5-diCQA	**4848 ± 251**	4568 ± 237	**3210 ± 170**	3932 ± 206	4128 ± 206	4716 ± 245	4190 ± 218
∑	63807	63354	62731	61958	65481	70659	60430

Table S12. Statistical analysis of the effect of assisted extraction techniques and their characteristic variables/conditions on the assessment of the quantity of chlorogenic acids in artichoke heart extracts (Fcrit = 7.708).

**Compound shortcut**	**UASE/SSDM**				**MASE/SSDM**				**PLE/SSDM**			
	**37 kHz**		**80 kHz**		**100%**		**50°C**		**100°C**		**150°C**	
	F	***p***	***F***	***p***	***F***	***p***	***F***	***p***	***F***	***p***	***F***	***p***
1-CQA	-	-	-	-	-	-	-	-	-	-	-	-
3-CQA	0.55	$5\cdot10^{-01}$	0.49	$5\cdot10^{-01}$	**8.75**	$4\cdot10^{-02}$	0.11	$8\cdot10^{-01}$	4.48	$1\cdot10^{-01}$	**36.31**	$4\cdot10^{-03}$
5-CQA	**9.26**	$4\cdot10^{-02}$	7.51	$5\cdot10^{-02}$	**49.90**	$2\cdot10^{-03}$	**21.97**	$1\cdot10^{-02}$	**14.52**	$2\cdot10^{-02}$	**23.30**	$8\cdot10^{-03}$
4-CQA	**61.69**	$1\cdot10^{-03}$	**38.51**	$3\cdot10^{-03}$	1.24	$3\cdot10^{-01}$	1.36	$3\cdot10^{-01}$	1.94	$2\cdot10^{-01}$	**14.93**	$2\cdot10^{-02}$
cis-5-CQA	0.04	$8\cdot10^{-01}$	3.79	$1\cdot10^{-01}$	**8.97**	$4\cdot10^{-02}$	**30.19**	$5\cdot10^{-03}$	**67.22**	$1\cdot10^{-03}$	**230.32**	$1\cdot10^{-04}$
1,3-diCQA	1.12	$3\cdot10^{-01}$	0.86	$4\cdot10^{-01}$	4.12	$1\cdot10^{-01}$	**18.56**	$1\cdot10^{-02}$	2.09	$2\cdot10^{-01}$	0.46	$5\cdot10^{-01}$
CA	-	-	-	-	-	-	-	-	-	-	-	-
CME	-	-	-	-	-	-	-	-	-	-	-	-
1,4-diCQA	-	-	-	-	-	-	-	-	-	-	-	-
3,4-diCQA	1.91	$2\cdot10^{-01}$	0.34	$6\cdot10^{-01}$	**25.72**	$7\cdot10^{-03}$	1.48	$3\cdot10^{-01}$	0.05	$8\cdot10^{-01}$	7.27	$5\cdot10^{-02}$
1,5-diCQA	**13.06**	$2\cdot10^{-02}$	6.96	$5\cdot10^{-02}$	**42.82**	$3\cdot10^{-03}$	3.53	$1\cdot10^{-01}$	0.13	$7\cdot10^{-01}$	**8.60**	$4\cdot10^{-02}$
3,5-diCQA	-	-	-	-	-	-	-	-	-	-	-	-
4,5-diCQA	**11.73**	$2\cdot10^{-02}$	4.12	$1\cdot10^{-01}$	**37.63**	$4\cdot10^{-03}$	2.21	$2\cdot10^{-01}$	0.12	$7\cdot10^{-01}$	7.71	$5\cdot10^{-02}$

Table S13. Quantity of chlorogenic acids and their derivatives estimated in coltsfoot (*Tussilago farfara* L.) herb extracts obtained using assisted extraction techniques and SSDM (expressed in μg/g ± SD).

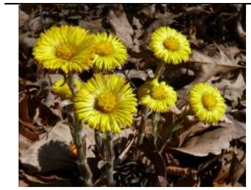

Compound shortcut	UASE 37 kHz	UASE 80 kHz	MASE 100%	PLE 50°C	PLE 100°C	PLE 150°C	SSDM
1-CQA	-	-	-	-	-	-	-
3-CQA	611 ± 44	487 ± 51	677 ± 54	569 ± 46	625 ± 50	**886 ± 71**	635 ± 51
5-CQA	**8868 ± 709**	5285 ± 423	**9687 ± 1792**	5307 ± 425	**7594 ± 608**	**10125 ± 810**	5535 ± 886
4-CQA	**1759 ± 89**	1134 ± 172	**1775 ± 72**	1122 ± 128	1143 ± 78	**2083 ± 147**	1183 ± 95
cis-5-CQA	-	-	-	-	-	-	-
1,3-diCQA	-	-	-	-	-	-	-
**CA**	**-**	**-**	**-**	**-**	**-**	**-**	**-**
CME	-	-	-	-	-	-	-
1,4-diCQA	-	-	-	-	-	-	-
3,4-diCQA	22488 ± 1939	20141 ± 1361	**27938 ± 1485**	20069 ± 1980	17763 ± 1421	18518 ± 1481	20306 ± 1624
1,5-diCQA	**5177 ± 1318**	5096 ± 318	6002 ± 859	5004 ± 538	**4364 ± 269**	5146 ± 286	5116 ± 319
3,5-diCQA	**1594 ± 95**	1684 ± 144	2134 ± 171	1430 ± 188	1521 ± 122	2055 ± 164	1850 ± 79
4,5-diCQA	4120 ± 329	3972 ± 408	4293 ± 625	37238 ± 400	3365 ± 349	3574 ± 412	3982 ± 552
∑	**44615**	**37798**	**52506**	**37238**	**36375**	**42388**	**38608**

Table S14. Statistical analysis of the effect of assisted extraction techniques and their characteristic variables/conditions on the assessment of the quantity of chlorogenic acids in coltsfoot extracts (Fcrit = 7.708).

**Compound shortcut**	**UASE/SSDM**				**MASE/SSDM**				**PLE/SSM**			
	**37 kHz**		**80 kHz**		**100%**		**50°C**		**100°C**		**150°C**	
	F	***p***	***F***	***p***	***F***	***p***	***F***	***p***	***F***	***p***	***F***	***p***
1-CQA												
3-CQA	0.41	$6\cdot10^{-01}$	5.14	$9\cdot10^{-02}$	0.23	$6\cdot10^{-01}$	2.83	$2\cdot10^{-01}$	2.88	$2\cdot10^{-01}$	**24.78**	$8\cdot10^{-03}$
5-CQA	**25.88**	$7\cdot10^{-03}$	0.19	$7\cdot10^{-01}$	**12.95**	$2\cdot10^{-02}$	0.16	$7\cdot10^{-01}$	**11.03**	$3\cdot10^{-02}$	**43.88**	$3\cdot10^{-03}$
4-CQA	**58.85**	$2\cdot10^{-03}$	0.19	$6\cdot10^{-01}$	**74.58**	$9\cdot10^{-03}$	0.44	$5\cdot10^{-01}$	0.32	$6\cdot10^{-01}$	**79.80**	$8\cdot10^{-04}$
cis-5-CQA												
1,3-diCQA												
CA												
CME		-				-				-		-
1,4-diCQA												
3,4-diCQA	2.23	$2\cdot10^{-01}$	0.02	$9\cdot10^{-01}$	**36.07**	$4\cdot10^{-03}$	0.03	$9\cdot10^{-01}$	4.16	$1\cdot10^{-01}$	1.98	$2\cdot10^{-01}$
1,5-diCQA	**13.02**	$2\cdot10^{-02}$	3.10	$1\cdot10^{-01}$	6.80	$6\cdot10^{-02}$	**12.83**	$2\cdot10^{-02}$	**15.44**	$2\cdot10^{-02}$	3.77	$1\cdot10^{-01}$
3,5-diCQA	**29.56**	$5\cdot10^{-03}$	0.01	$9\cdot10^{-01}$	0.34	$6\cdot10^{-01}$	0.45	$5\cdot10^{-01}$	6.56	$6\cdot10^{-02}$	2.72	$2\cdot10^{-02}$
4,5-diCQA	0.03	$8\cdot10^{-01}$	0.01	$9\cdot10^{-01}$	3.38	$1\cdot10^{-01}$	0.08	$8\cdot10^{-01}$	3.98	$1\cdot10^{-01}$	0.01	$9\cdot10^{-01}$

Table S15. Quantity of chlorogenic acids and their derivatives estimated in chamomile (*Matricaria* L.) flower extracts obtained by assisted extraction techniques and SSDM (expressed in µg/g ± SD).

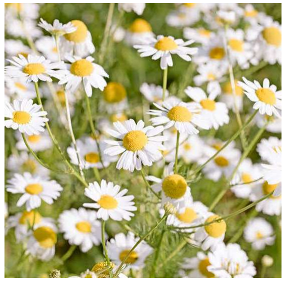

Compound shortcut	UASE 37 kHz	UASE 80 kHz	MASE 100%	PLE 50ºC	PLE 100ºC	PLE 150ºC	SSDM
1-CQA	-	-	-	-	-	-	-
3-CQA	**203 ± 20**	**206 ± 10**	271 ± 18	**205 ± 6**	244 ± 22	274 ± 15	264 ± 13
5-CQA	**1841 ± 102**	**1820 ± 97**	**2785 ± 154**	**1711 ± 75**	2106 ± 115	2239 ± 122	2134 ± 98
4-CQA	**283 ± 7**	**278 ± 14**	**470 ± 23**	**284 ± 14**	**324 ± 16**	383 ± 19	383 ± 11
cis-5-CQA	-	-	-	-	-	-	-
1,3-diCQA	-	-	-	-	-	-	-
CA	1931 ± 97	**1329 ± 132**	2073 ± 104	2040 ± 102	2324 ± 116	**2391 ± 120**	2102 ± 105
CME	**259 ± 45**	**205 ± 23**	**375 ± 19**	-	-	-	-
1,4-diCQA	-	-	-	-	-	-	-
3,4-diCQA	1553 ± 105	1529 ± 76	1612 ± 100	**1899 ± 110**	**1949 ± 97**	**2003 ± 126**	1397 ± 89
1,5-diCQA* 3,5-diCQA*	7066 ± 415	7024 ± 239	7354 ± 368	6910 ± 276	**7429 ± 420**	7006 ± 351	6538 ± 287
4,5-diCQA	1686 ± 75	1504 ± 105	1876 ± 94	1766 ± 124	**1931 ± 127**	**2019 ± 215**	1470 ± 168
∑	**14821**	**13895**	**16815**	**14816**	**16308**	**16315**	**14290**

Table S16. Statistical analysis of the effect of assisted extraction techniques and their characteristic variables/conditions on the assessment of the quantity of chlorogenic acids in chamomile extracts (Fcrit = 7.708).

**Compound shortcut**	**UASE/SSDM**				**MASE/SSDM**		**PLE/SSM**					
	**37 kHz**		**80 kHz**		**100%**		**50°C**		**100°C**		**150°C**	
	F	***p***	***F***	***p***	***F***	***p***	***F***	***p***	***F***	***p***	***F***	***p***
1-CQA	-	-	-	-	-	-	-	-	-	-	-	-
3-CQA	**19.84**	$1\cdot10^{-02}$	**37.27**	$3.6\cdot10^{-03}$	0.25	$6.4\cdot10^{-01}$	**50.95**	$2\cdot10^{-03}$	1.82	$2\cdot10^{-01}$	0.66	$4.6\cdot10^{-01}$
5-CQA	**12.87**	$2.3\cdot10^{-02}$	**15.53**	$1.7\cdot10^{-02}$	**38.15**	$3.5\cdot10^{-03}$	**35.22**	$4.0\cdot10^{-03}$	0.09	$7.7\cdot10^{-01}$	1.35	$3\cdot10^{-01}$
4-CQA	**179.6**	$1.7\cdot10^{-04}$	**107.11**	$5\cdot10^{-04}$	**33.39**	$4\cdot10^{-03}$	**91.60**	$6\cdot10^{-04}$	**27.29**	$6.4\cdot10^{-03}$	0.00	$9\cdot10^{-01}$
cis-5-CQA	-	-	-	-	-	-	-	-	-	-	-	-
1,3-diCQA	-	-	-	-	-	-	-	-	-	-	-	-
CA	4.35	$1.1\cdot10^{-01}$	**63.06**	$1.4\cdot10^{-03}$	0.12	$7.4\cdot10^{-01}$	0.55	$4.9\cdot10^{-01}$	5.97	$7\cdot10^{-02}$	**9.8**	$3.4\cdot10^{-02}$
CME	**99.1**	$5.7\cdot10^{-04}$	**237.86**	$1\cdot10^{-04}$	**1200**	$4.1\cdot10^{-06}$	-	-	-	-	-	-
1,4-diCQA	-	-	-	-	-	-	-	-	-	-	-	-
3,4-diCQA	3.83	$1.2\cdot10^{-01}$	3.78	$1.2\cdot10^{-02}$	**7.75**	$5\cdot10^{-02}$	**37.63**	$3.5\cdot10^{-03}$	**52.2**	$2\cdot10^{-03}$	**46.3**	$2\cdot10^{-03}$
1,5-diCQA 3,5-diCQA	3.27	$1.4\cdot10^{-01}$	5.06	$9\cdot10^{-02}$	**9.14**	$4\cdot10^{-02}$	2.61	$2\cdot10^{-01}$	**9.2**	$4\cdot10^{-02}$	3.2	$1\cdot10^{-01}$
4,5-diCQA	4.14	$1\cdot10^{-01}$	0.9	$8\cdot10^{-01}$	**13.4**	$2\cdot10^{-02}$	6.04	$7\cdot10^{-02}$	**14.4**	$2\cdot10^{-02}$	**12.1**	$2\cdot10^{-02}$

Table S17. Quantity of chlorogenic acids and their derivatives estimated in tansy (*Tanacetum*L.)flower extracts obtained by assisted extraction techniques and SSDM (expressed in μg/g ± SD).

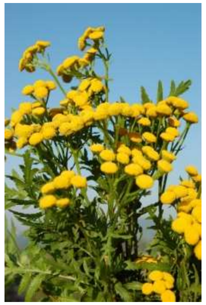

**Compound shortcut**	**UASE** 37 kHz	80 kHz	**MASE** 100%	**PLE** 50°C	100°C	150°C	**SSDM**
1-CQA	**195 ± 18**	**155 ± 15**	**186 ± 18**	83 ± 8	105 ± 10	**167 ± 16**	99 ± 9
3-CQA	474 ± 45	400 ± 38	502 ± 47	483 ± 45	570 ± 54	**705 ±66**	478 ± 45
5-CQA	7139 ± 671	6569 ± 620	7144 ± 672	6420 ± 603	7830 ± 736	6142 ± 577	6478 ± 609
4-CQA	**558 ± 52**	**391 ± 37**	**432 ± 41**	524 ± 49	605 ± 57	**655 ± 62**	714 ± 67
cis-5-CQA	-	-	-	-	-	-	-
1,3-diCQA	-	-	-	-	-	-	-
CA	-	-	-	-	-	-	-
CME	-	-	-	-	-	-	-
1,4-diCQA	-	-	-	-	-	-	-
3,4-diCQA	1685 ± 158	1748 ± 164	1299 ± 122	1702 ± 160	1542 ± 145	**2557 ± 240**	1461 ± 137
1,5-diCQA*<br>3,5-diCQA*	16902 ± 1589	16037 ± 1507	15599 ± 1466	15267 ± 1435	16361 ± 1538	**10704 ± 1006**	15219 ± 1431
4,5-diCQA	5947 ± 559	5425 ± 510	5076 ± 477	5468 ± 514	**6563 ± 617**	**7564 ± 711**	4881 ± 459
∑	**32900**	**30724**	**30238**	**29948**	**33575**	**28494**	**29331**

*inseparablepeaks

Table S18. Statistical analysis of the effect of assisted extraction techniques and their characteristic variables/conditions on the assessment of the quantity of chlorogenic acids in tansy extracts (Fcrit = 7.708).

**Compound shortcut**	**UASE/SSDM**				**MASE/SSDM**				**PLE/SSM**			
	**37 kHz**		**80 kHz**		**100%**		**50°C**		**100°C**		**150°C**	
	F	***p***	***F***	***p***	***F***	***p***	***F***	***p***	***F***	***p***	***F***	***p***
1-CQA	**64.44**	$1\cdot10^{-03}$	**30.8**	$5\cdot10^{-03}$	**57.42**	$2\cdot10^{-03}$	5.6	$8\cdot10^{-02}$	0.5	$5\cdot10^{-01}$	**41.2**	$3\cdot10^{-03}$
3-CQA	0.01	$9\cdot10^{-01}$	5.4	$8\cdot10^{-02}$	0.39	$5\cdot10^{-01}$	0.02	$9\cdot10^{-01}$	5.2	$8\cdot10^{-02}$	**24.1**	$8\cdot10^{-03}$
5-CQA	1.59	$3\cdot10^{-01}$	0.03	$9\cdot10^{-01}$	1.62	$3\cdot10^{-01}$	0.01	$9\cdot10^{-01}$	6.01	$7\cdot10^{-02}$	0.48	$5\cdot10^{-01}$
4-CQA	**10.04**	$3\cdot10^{-02}$	**53.51**	$2\cdot10^{-03}$	**38.73**	$3\cdot10^{-03}$	3.91	$1\cdot10^{-01}$	0.48	$5\cdot10^{-01}$	**17.01**	$2\cdot10^{-03}$
cis-5-CQA	-	-	-	-	-	-	-	-	-	-	-	-
1,3-diCQA	-	-	-	-	-	-	-	-	-	-	-	-
CA	-	-	-	-	-	-	-	-	-	-	-	-
CME	-	-	-	-	-	-	-	-	-	-	-	-
1,4-diCQA	-	-	-	-	-	-	-	-	-	-	-	-
3,4-diCQA	3.4	$1\cdot10^{-01}$	5.4	$8\cdot10^{-02}$	2.32	$2\cdot10^{-01}$	3.91	$1\cdot10^{-01}$	0.48	$5\cdot10^{-01}$	**47.01**	$2\cdot10^{-02}$
1,5-diCQA<br>3,5-diCQA	1.85	$2\cdot10^{-01}$	0.46	$5\cdot10^{-01}$	0.10	$8\cdot10^{-01}$	0.00	$1\cdot10^{-01}$	0.90	$4\cdot10^{-01}$	**20.00**	$1\cdot10^{-02}$
4,5-diCQA	6.51	$6\cdot10^{-02}$	1.88	$2\cdot10^{-01}$	0.26	$6\cdot10^{-01}$	2.17	$2\cdot10^{-01}$	**14.36**	$2\cdot10^{-02}$	**30.17**	$5\cdot10^{-03}$

Table S19. Quantity of chlorogenic acids and their derivatives estimated in yarrow (*Achillea* L.) flower extracts obtained using assisted extraction techniques and SSDM (expressed in µg/g ± SD).

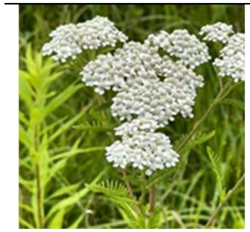

**Compound shortcut**	**UASE** 37 kHz	**UASE** 80 kHz	**MASE** 100%	**PLE** 50°C	**PLE** 100°C	**PLE** 150°C	**SSDM**
1-CQA	**463 ± 21**	**274 ± 13**	**158 ± 7**	**255 ± 12**	157 ± 7	**288 ± 13**	215 ± 10
3-CQA	**327 ± 15**	443 ± 20	**465 ± 21**	384 ± 18	824 ± 38	**731 ± 34**	418 ± 19
5-CQA	7602 ± 350	6607 ± 304	7293 ± 335	6375 ± 293	8164 ± 368	**8234 ± 189**	7019 ± 323
4-CQA	437 ± 20	441 ± 20	**525 ± 24**	396 ± 18	568 ± 52	**507 ± 47**	431 ± 20
cis-5-CQA	-	-	-	-	-	-	-
1,3-diCQA	-	-	-	-	-	-	-
CA	-	-	-	-	-	-	-
CME	-	-	-	-	-	-	-
1,4-diCQA	**359 ±17**	**279 ± 13**	**368 ± 17**	**158 ± 7**	299 ± 14	**311 ± 14**	246 ± 11
3,4-diCQA	**1179 ± 54**	957 ± 44	**1180 ± 54**	**1129 ± 52**	1623 ± 75	**2000 ± 92**	965 ± 44
1,5-diCQA*<br>3,5-diCQA*	**11833 ± 544**	9596 ± 441	10305 ± 438	9523 ± 501	10886 ± 388	8427 ± 460	10008 ± 1431
4,5-diCQA	**2508 ± 115**	2098 ± 97	2168 ± 98	2123 ± 170	3685 ± 232	**5038 ± 98**	2126 ± 459
∑	**24706**	**20695**	**22463**	**20343**	**26206**	**25536**	**21428**

Table S20. Statistical analysis of the effect of assisted extraction techniques and their characteristic variables/conditions on the assessment of the quantity of chlorogenic acids in yarrow extracts (Fcrit = 7.708).

**Compound shortcut**	**UASE/SSDM**				**MASE/SSDM**				**PLE/SSM**			
	**37 kHz**		**80 kHz**		**100%**		**50°C**		**100°C**		**150°C**	
	F	***p***	***F***	***p***	***F***	***p***	***F***	***p***	***F***	***p***	***F***	***p***
1-CQA	**335.48**	$5.2\cdot10^{-05}$	**41.81**	$3\cdot10^{-03}$	**64.79**	$1\cdot10^{-03}$	**20.56**	$1\cdot10^{-02}$	**65.30**	$1\cdot10^{-02}$	**58.72**	$2\cdot10^{-03}$
3-CQA	**41.73**	$3\cdot10^{-03}$	2.24	$2\cdot10^{-01}$	**7.89**	$5\cdot10^{-02}$	5.17	$8\cdot10^{-02}$	**273.03**	$8\cdot10^{-05}$	**195.62**	$2\cdot10^{-04}$
5-CQA	4.49	$1\cdot10^{-02}$	2.59	$2\cdot10^{-01}$	1.04	$4\cdot10^{-01}$	6.54	$6\cdot10^{-02}$	**21.79**	$9\cdot10^{-03}$	**17.88**	$1\cdot10^{-02}$
4-CQA	0.11	$7\cdot10^{-01}$	0.37	$6\cdot10^{-02}$	**27.08**	$6\cdot10^{-03}$	5.06	$9\cdot10^{-02}$	**477.04**	$3\cdot10^{-05}$	**395.64**	$4\cdot10^{-05}$
cis-5-CQA	-	-	-	-	-	-	-	-	-	-	-	-
1,3-diCQA	-	-	-	-	-	-	-	-	-	-	-	-
CA	-	-	-	-	-	-	-	-	-	-	-	-
CME	-	-	-	-	-	-	-	-	-	-	-	-
1,4-diCQA	**95.76**	$6\cdot10^{-04}$	**11.12**	$3\cdot10^{-02}$	**108.85**	$5\cdot10^{-04}$	**129.15**	$3\cdot10^{-04}$	**26.44**	$7\cdot10^{-03}$	**38.70**	$3\cdot10^{-03}$
3,4-diCQA	**27.98**	$6\cdot10^{-03}$	0.04	$8\cdot10^{-01}$	**28.29**	$6\cdot10^{-03}$	**17.38**	$1\cdot10^{-02}$	**172.38**	$2\cdot10^{-04}$	**308.41**	$6\cdot10^{-05}$
1,5-diCQA 3,5-diCQA	**19.65**	$1\cdot10^{-02}$	1.25	$3\cdot10^{-01}$	0.60	$5\cdot10^{-01}$	1.74	$3\cdot10^{-01}$	4.99	$9\cdot10^{-02}$	**20.71**	$1\cdot10^{-02}$
4,5-diCQA	**19.05**	$1\cdot10^{-02}$	0.12	$7\cdot10^{-01}$	0.27	$6\cdot10^{-01}$	0.00	$9\cdot10^{-01}$	**190.23**	$2\cdot10^{-04}$	**402.00**	$4\cdot10^{-05}$

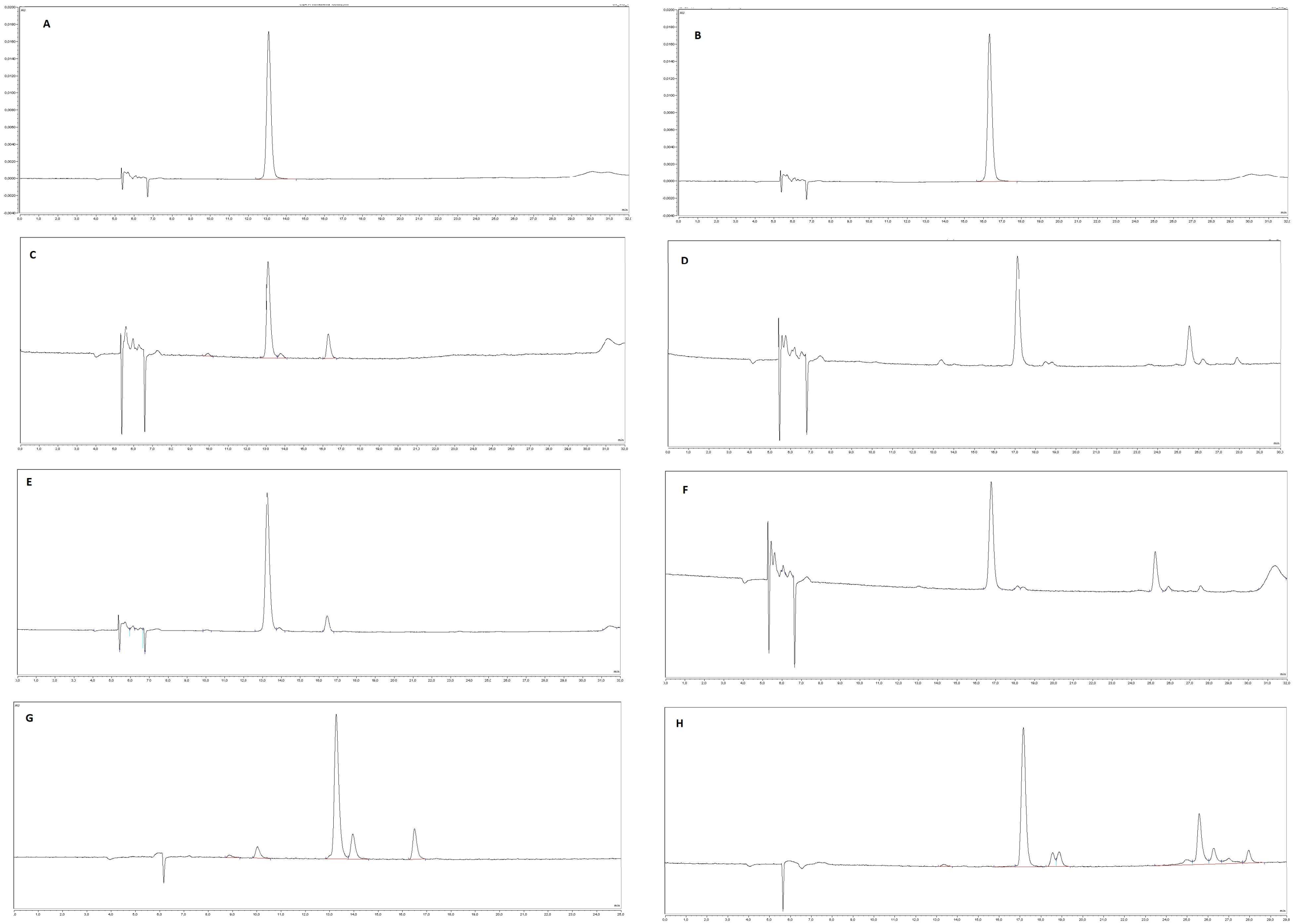

Figure S4. System chromatograms, for data from Figure 2, of 5-CQA (A) and 1,3-diCQA (B) standard solutions without and after simulated MASE extraction at 50 and 100% generator power for 30 and 60 min, recorded at 330 nm (detailed description in the text)

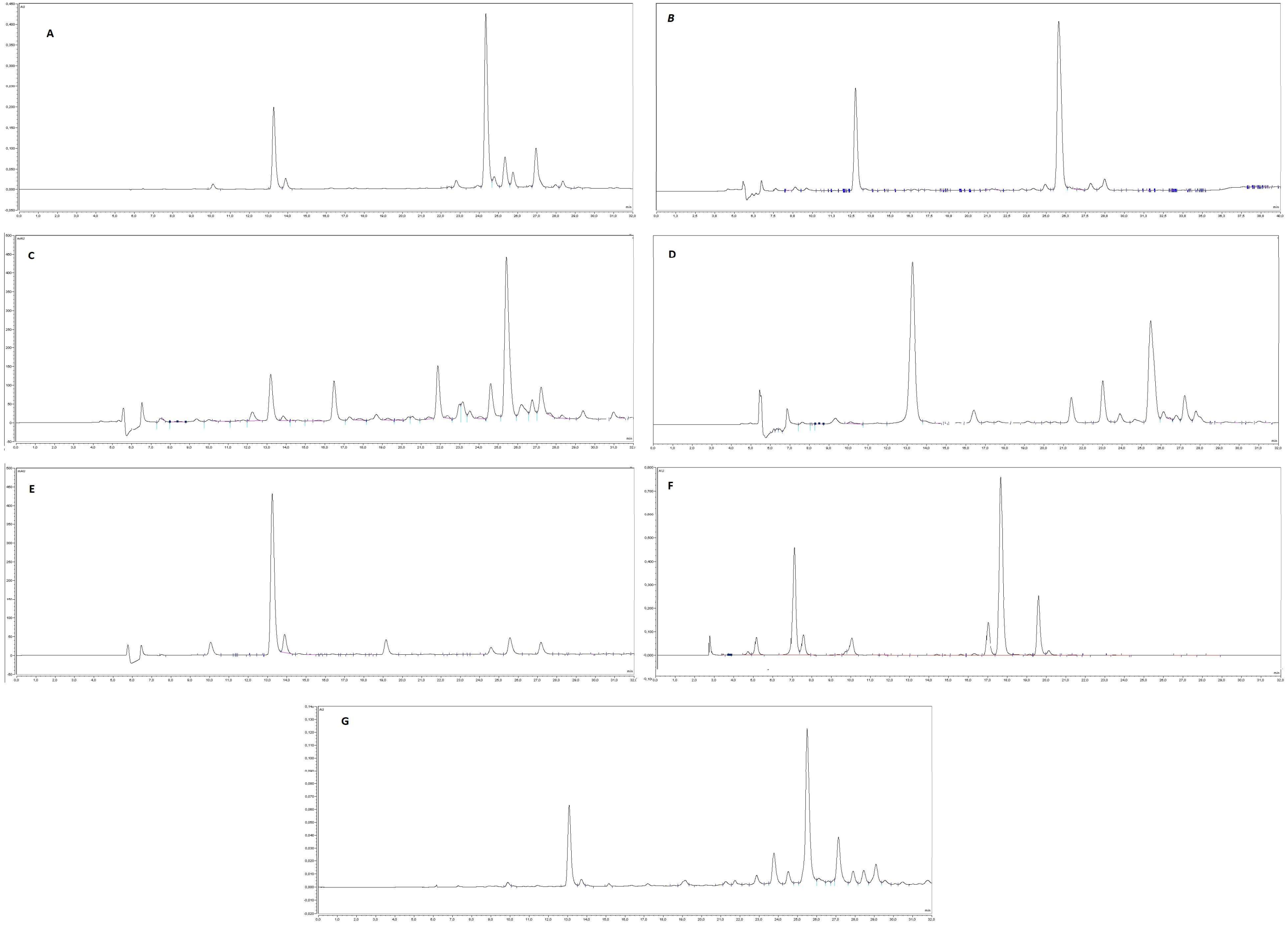

Figure S5. System chromatograms, for data from Figure 6, of extracts of coltsfoot (A), yarrow (B), chamomile (C), artichoke leaves (D), green coffee beans (E), artichoke bud “heart” (F), and tansy herb (G) obtained by UASE at 37kHz.
